# Dual RNA Sequencing Reveals Key Events When Different *Giardia* Life Cycle Stages Interact With Human Intestinal Epithelial Cells *In Vitro*

**DOI:** 10.3389/fcimb.2022.862211

**Published:** 2022-04-27

**Authors:** Laura Rojas, Jana Grüttner, Showgy Ma’ayeh, Feifei Xu, Staffan G. Svärd

**Affiliations:** ^1^ Department of Cell and Molecular Biology, Uppsala University, Uppsala, Sweden; ^2^ Olink Proteomics, Uppsala, Sweden; ^3^ SciLifeLab, Uppsala University, Uppsala, Sweden

**Keywords:** diarrhea, dual RNAseq, small intestinal epithelial cells, protozoa, differentiation

## Abstract

*Giardia intestinalis* is a protozoan parasite causing diarrheal disease, giardiasis, after extracellular infection of humans and other mammals’ intestinal epithelial cells (IECs) of the upper small intestine. The parasite has two main life cycle stages: replicative trophozoites and transmissive cysts. Differentiating parasites (encysting cells) and trophozoites have recently been shown to be present in the same regions of the upper small intestine, whereas most mature cysts are found further down in the intestinal system. To learn more about host-parasite interactions during *Giardia* infections, we used an *in vitro* model of the parasite’s interaction with host IECs (differentiated Caco-2 cells) and *Giardia* WB trophozoites, early encysting cells (7 h), and cysts. Dual RNA sequencing (Dual RNAseq) was used to identify differentially expressed genes (DEGs) in both *Giardia* and the IECs, which might relate to establishing infection and disease induction. In the human cells, the largest gene expression changes were found in immune and MAPK signaling, transcriptional regulation, apoptosis, cholesterol metabolism and oxidative stress. The different life cycle stages of *Giardia* induced a core of similar DEGs but at different levels and there are many life cycle stage-specific DEGs. The metabolic protein PCK1, the transcription factors HES7, HEY1 and JUN, the peptide hormone CCK and the mucins MUC2 and MUC5A are up-regulated in the IECs by trophozoites but not cysts. Cysts specifically induce the chemokines CCL4L2, CCL5 and CXCL5, the signaling protein TRKA and the anti-bacterial protein WFDC12. The parasite, in turn, up-regulated a large number of hypothetical genes, high cysteine membrane proteins (HCMPs) and oxidative stress response genes. Early encysting cells have unique DEGs compared to trophozoites (e.g. several uniquely up-regulated HCMPs) and interaction of these cells with IECs affected the encystation process. Our data show that different life cycle stages of *Giardia* induce different gene expression responses in the host cells and that the IECs in turn differentially affect the gene expression in trophozoites and early encysting cells. This life cycle stage-specific host-parasite cross-talk is an important aspect to consider during further studies of *Giardia*’s molecular pathogenesis.

## Introduction

Diarrheal diseases play a major role in disease burden worldwide, being a leading cause of death and malnutrition in children <5 years of age (Kotloff et al., 2013). *Giardia intestinalis* is a unicellular protozoan parasite that infects humans and other mammals, producing a diarrheal infection called giardiasis (Adam, 2021). Giardiasis has a significantly higher impact in low-income countries and regions with poor water quality, sanitation, and food safety (Pires et al., 2015; Ryan et al., 2019).

*Giardia* alternates its life-cycle between two main stages; the disease-causing trophozoite and the environmentally resistant cysts (Adam, 2021). Trophozoites are non-invasive and establish infection in the upper small intestine. Even though giardiasis does not produce a severe inflammatory response, it has been associated with intestinal epithelial cell (IEC) damage. This damage leads to villus shortening, enterocyte apoptosis, and intestinal barrier dysfunction (Halliez and Buret, 2013), resulting in malabsorption of water, electrolytes, glucose, and maldigestion (Scott et al., 2000; Solaymani-Mohammadi & Singer, 2011). Cyst formation is an essential factor for the survival and establishment of the parasite in a new host, and this process has been believed to only occur in the lower intestine. However, earlier *in vivo* experiments in gerbils and recent experiments in mice and gerbils using recombinant parasites have shown that encysting cells are also found in the small intestine, in the same regions as trophozoites in specific intestinal foci (Campbell & Faubert, 1994; Barash et al., 2017; Pham et al., 2017). Most mature cysts were observed in the last section of the small intestine and the colon (Campbell & Faubert, 1994). Encysting parasites were also identified together with trophozoites in the upper small intestine of mice infected by the murine parasite *Giardia muris* (Xu et al., 2020b). These observations suggest the presence of different life cycle stages of *Giardia* in the same intestinal foci.

Earlier host-*Giardia* interaction studies have combined *Giardia* trophozoites from different assemblages (A and B) with different intestinal cells lines (Roxström-Lindquist et al., 2005; Ringqvist et al., 2011; Ma’ayeh & Brook-Carter, 2012; Ferella et al., 2014; Peirasmaki et al., 2020), and recently a study used small intestinal enteroids as host cells (Holthaus et al., 2021). However, none of these studies analyzed the interaction of the parasite in other life cycle stages than trophozoites.

Transcriptomes are often analyzed in a first attempt to understand molecular, cellular, and organismic events during host-parasite interactions. A comprehensive profile of RNA expression can be obtained using high-throughput sequencing of cDNA from reverse-transcribed expressed RNA. Such RNA sequencing (RNAseq) provides high technical accuracy at a reasonable cost, making it the current method for transcriptomics. Furthermore, RNAseq can simultaneously assess host and pathogen transcriptomes if RNA from both organisms is contained in a sample in an infection experiment (Dual RNAseq). In this study, we used *Giardia* isolate WB-C6 parasites from different life cycle stages (trophozoites, 7 h encysting cells, and cysts) combined with human intestinal epithelial cells (differentiated Caco-2 cells), and Dual RNAseq to identify differentially expressed *Giardia* and host genes during interactions *in vitro*. Our data show that different life-cycle stages of *Giardia* induce different gene expression responses in the host cells and this can be an important factor during *Giardia* infections.

## Material And Methods

### Cell Culture

Human colon adenocarcinoma cells (Caco-2, clone TC7, passage no. 6-9) were cultured for the interaction experiments until a differentiated state was obtained as earlier described (Roxström-Lindquist et al., 2005). The Caco-2 cells were cultured in 25 cm^2^ tissue culture flasks (T25) or in 6- or 12-well plates (Corning, cat no. 3471 and 3513) in Dulbecco’s modified Eagle’s medium (DMEM) (Sigma-Aldrich) supplemented with 10% heat-inactivated fetal bovine serum (FBS, Thermo Fisher Scientific, cat no. 10100147), 2 mM GlutaMAX (Gibco, Thermo Fisher Scientific, MA, USA), 1x MEM non-essential amino acid solution (Sigma-Aldrich, MO, USA) and 1x of penicillin-streptomycin 100x solution (10,000 units penicillin and 10 mg streptomycin/ml) (Sigma-Aldrich, MO, USA). Culture flasks and plates were incubated in a humidified incubator (10% CO_2_ and 37°C) for 21 days until differentiation, during which the media was changed twice weekly. The differentiation status of the Caco-2 cells was checked by microscopy and expression of aminopeptidase N and alkaline phosphatase as earlier described (Roxström-Lindquist et al., 2005).

*Giardia intestinalis* isolate WB, clone C6 (ATCC 30957) was used in the experiments. Trophozoites were cultured in 50 ml Falcon tubes filled with TYDK media (Keister, 1983) supplemented with 10% heat-inactivated bovine serum (Gibco, Thermo Fisher MA, USA). All tubes were incubated at 37°C until reaching 80% confluence. The trophozoites were used in interaction experiments or to induce encystation for 7 hours or to produce mature cysts following the Uppsala protocol (Einarsson et al., 2016). In order to get cysts a 32 h encysting population was centrifugated to remove the encystation medium, the cell pellet was resuspended in water and washed 3 times before being water-treated for 72 h at 4°C. After the water-treatment the cells were washed again 2 times before being added to the IEC interaction. Unless otherwise stated, all chemicals used in the TYDK and encystation media were purchased from Sigma-Aldrich.

### Host-Parasite Interactions

At the beginning of the experiment, trophozoites, encysting cells, cysts and human cell cultures (grown in T25 flasks for RNA sequencing and in 6-well plates for ELISAs) were washed twice with warm PBS (37°C) and replenished with fresh DMEM with 10% HI-FBS (50 ml for parasite cultures and 9 ml or 2.5 ml for human cell cultures (grown in T25 flask and 6-well plates, respectively. Incubation of the cells in DMEM before the experiment was deemed necessary to reduce shifts in gene transcription due to media change (i.e., from TYDK to DMEM). Initially, all cultures were incubated in a humidified tissue culture incubator for 2 h at 37°C, after which trophozoites were processed for addition to IECs. This involved incubating culture tubes on ice (10 min) for detachment, counting (Neubauer Chamber slide), pelleting (centrifugation; 7 min, 750 x g, and 4°C), resuspension in DMEM, and finally addition to human cells at a concentration of 1.2x10^6^cells/cm^2^ (1ml of 3x10^7^ cells/ml for T25 flasks and 500µl of 2.28x10^7^ cells/ml for 6-well plates). Next, parasites were incubated with IECs for 1.5, 3, and 4.5 h for RNA sequencing samples and 6 h for ELISA samples (10% CO_2_ at 37°C). Control parasites and human cells were incubated in the same conditions without addition of the other cell type. At the end of each time point, the interactions media were removed from the flasks. The co-cultures for RNA sequencing analysis were lysed directly in 1.5 ml of lysis buffer for RNA extraction for trophozoites and 1 ml Trizol for encysting and cysts interactions. The lysis buffer was included in the PureLink RNA Mini Kit (Ambion, Thermo Fisher Scientific), and beta-mercaptoethanol was added directly prior to cell lysis. For the control, trophozoites, treated as above, were pelleted and lysed directly in the RNA lysis buffer. Encysting cells and cysts were pelleted and lysed in Trizol. All samples were collected in RNase-free Eppendorf tubes and frozen immediately in dry ice. Samples were kept at – 80°C until RNA extraction.

### RNA Extraction, Library Preparation, and RNA Sequencing

The samples collected for the trophozoites interaction were processed according to the instructions provided in the PureLink RNA Mini Kit (Ambion, Thermo Fisher Scientific). While the samples collected for the interaction of encysting and cysts interaction were extracted with Trizol as earlier described (Franzén et al., 2013). A DNase I treatment step (PureLink DNase Set, Ambion, Thermo Fisher Scientific) was performed during RNA extraction to remove genomic DNA before eluting the RNA. RNA quality was assessed by evaluating the 260/280 and 260/230 ratios (NanoDrop 1000 Spectrometer, Thermo Fisher Scientific) and electrophoresing the samples (500 ng) on a 1.5% Tris-Borate-EDTA (TBE) agarose gel containing 20 mM of guanidium isothiocyanate (GITC). Sequencing libraries were prepared from 500 ng of total RNA using the TruSeq stranded mRNA library preparation kit (cat no. RS-122-2101/2102, Illumina Inc.), which included a polyA selection step. Library preparations were performed following the manufacturers’ protocol (cat no. 15031047, Illumina Inc). The quality of prepared libraries was evaluated using a Fragment Analyzer from Advanced Analytical (AATI) using the DNF-910 kit, and they were quantified by qPCR using the Library quantification kit for Illumina (KAPA Biosystems) on a CFX384 Touch instrument (Bio-Rad) prior to cluster generation and sequencing. According to the manufacturer’s instructions, sequencing was carried out on an Illumina NovaSeq6000 instrument (NVCS v 1.3.0/RTA v3.3.3). De-multiplexing and conversion to FASTQ format were performed using the bcl2fastq2 (2.20.0.422) software provided by Illumina. Additional statistics on sequencing quality were compiled with an in-house script from the FASTQ-files, RTA, and BCL2FASTQ2 output files. RNA sequencing was performed at the SciLifeLab NovaSeq Sequencing Platform, Uppsala University, Sweden.

### Bioinformatic Analyses of RNA Sequencing Data

We constructed our own dual RNA sequencing pipeline for analyzing differentially transcribed genes of both *Giardia* and human genes during infection. All scripts for the bioinformatics analysis used here are available upon request. Briefly, STAR v020201 (Dobin et al., 2013) was used to map the RNA sequencing read counts to the *Giardia* WB reference genome (Xu et al., 2020a) and to the human genome (GRCh38.p12) and the parameter “–quantMode GeneCounts” was used to generate the raw counts per gene. All further data analysis was performed in R (version 4.0.1). The edgeR (v3.32.1) (Robinson et al., 2010) package was used for differential expression analysis with Quasi-likelihood (QL) *F*-test (glmQLFTest) to determine significant differential gene expression. Genes were considered significant when the false discovery rate (FDR) < 0.05. Reactome pathway and GO term (Molecular Function) enrichment analysis were performed on significant differentially expressed genes (with a log 2-fold change greater or smaller than 1, -1, respectively) using clusterProfiler (v3.18.1) (Yu et al., 2012). Hierarchical clustering of the samples (Complete method, Euclidean distance) was done on the same set of genes using the pvclust package (Suzuki and Shimodaira, 2006). Venn diagrams were made using the ggVennDiagram package (v 1.2.0) (Gao et al., 2021) and heatmaps were generated using the ComplexHeatmap package (v2.6.2) (Gu et al., 2016). Raw reads and processed raw counts per gene were deposited at Gene Expression Omnibus (GEO), available as accession ID GSE144004 for the trophozoite, GSE144001 for the encysting cells, and GSE172213for the cyst infection experiment. Data from the RNAseq analyses can be found in **Tables S1** **–** **S4**.

### Quantitative Polymerase Chain Reaction (qPCR)

High-quality intact RNA samples were processed for cDNA synthesis. First, 1 μg of each sample was treated with DNase I (Fermentas, ThermoFisher). Next, the DNA-free RNA was reverse transcribed according to the Revert Aid H Minus cDNA Synthesis instructions Kit (Thermo Fisher Scientific). Oligo-(dT)18 primers were used for cDNA synthesis. cDNA reaction mixtures were diluted (5 ng template per reaction) and used in the qPCR reactions together with 250 nM of each primer and the Maxima SYBR Green qPCR Master Mixes (Thermo Fisher Scientific). Reactions were set up in 20 μl volumes and run following the manufacturer’s instructions, including melt curve analysis at the end of the run. qPCRs were performed in a StepOne™Plus thermal cycler (Applied Biosystems, Carlsbad, USA). qPCR was performed on the genes *il8, cxcl1-3*, and *ccl20* using *gapdh* as endogenous control. All primers (Sigma Aldrich) were previously described by (Ma’ayeh et al., 2018). The fold change in gene expression was calculated using the δδCt method. Significant changes in RNA levels between treatments and controls were assessed using one-way analysis of variance (ANOVA) at α < 0.05 followed by the Dunnett’s multiple comparison test at P < 0.05.

### Evaluation of Cyst Formation in Different Media

For the media evaluation, trophozoites were grown up to 80% confluency in TYDK and then transferred to encystation medium for 7 h. After deattachment and centrifugation as above the cells were divided into different wells to evaluate the effect of the medium having 4 different groups: encysting cells in DMEM, interacting with Caco-2 cells in DMEM, in Uppsala encystation medium, and in TYDK. The interaction was kept for 30 h, and the final amount was evaluated by counting the number of viable cysts in the different media. This experiment was repeated 5 times.

### Western Blot Analyses

The interaction experiments with trophozoites and differentiated Caco-2 cells were repeated for different time points (0-10 h), and total cells were collected after being washed 3 times in warm PBS (37°C). In the last wash, cells were scraped off and pelleted by centrifugation (930 x *g*, 10 min, 4°C). The pellet was lysed in RIPA lysis buffer containing both Halt Protease and Phosphatase Inhibitor Cocktails (Thermo Fisher Scientific), followed by sonication for 30 s (50% amplitude) to reduce samples viscosity. The lysed cells were aliquoted and stored at -80°C until later use. Protein samples collected as described above were used for SDS-PAGE and Western blots. Protein samples (30 μg each) were electrophoresed (100 V) on AnykD gels (Biorad, CA, USA) and transferred onto a PVDF membrane for 90 min at 100 V (4°C). Blots were blocked with 5% non-fat dry milk in Tris Buffered Saline with 0.1% Tween-20 (TBST) for 1 h at room temperature (RT), followed by the addition of primary antibodies diluted in either 5% non-fat dry milk or 5% BSA in TBST and incubation overnight (4°C). The following primary antibodies targeting proteins were used: TTP (Cell Signaling Trechnology, MA, USA) Regnase-1/ZC3H12A (Thermo Fischer Scientific, MA, USA) and GAPDH (Sigma-Aldrich). The following day, blots were washed three times with TBST (5 min each) and incubated at RT with anti-mouse and anti-rabbit horseradish peroxidase-conjugated secondary antibodies (in 5% non-fat milk-TBST), developed using the Clarity™ ECL Western Blotting Substrate (BioRad) and viewed using a ChemiDoc Imaging System (BioRad). The TTP and Regnase1 primary antibodies were used at 1:1000 dilution. GAPDH primary antibody and anti-mouse and anti-rabbit secondary antibodies were used at a 1:10000 dilution. The above experiments were repeated three times to verify the results of Western blots. Densitometry for detected bands was performed using the Image J software (https://imagej.nih.gov/ij/, 1997-2016).

### ELISA

To measure the cytokines released by differentiated Caco-2 cells, ELISA was performed on the spent media collected from the interaction experiments. The assayed cytokines include CCL4, CCL5, CXCL2, and CXCL8. Kits used for cytokines measurement (DuoSet Human ELISA kits) were purchased from R&D Systems (MN, Canada), and measurements were performed following the manufacturer’s instructions. Absorbance reads from samples and standard curves were plotted in GraphPad Prism version 9.2.0 for Windows, GraphPad Software, San Diego, California USA, www.graphpad.com, using a four-parameter logistic ELISA curve fitting to obtain cytokine concentrations.

### Statistical Analysis

The data obtained from ELISAs were analyzed using a one-way analysis of variance (ANOVA) at α < 0.05, followed by Bonferroni comparisons (*P* < 0.05) to identify significant differences between the test groups versus control. In addition, the change in RNA levels between parasitized differentiated and proliferating Caco-2 cells was compared using a two-way ANOVA (α < 0.05) followed by Tukey’s pairwise comparisons (*P* < 0.05) to identify significant differences for each gene.

## Results

### Overall Gene Expression Changes During Caco-2 Interaction With Different Life Cycle Stages of *G. intestinalis*

In order to study how different stages of the *G. intestinalis* life cycle affect gene expression in human intestinal epithelial cells (IECs), we used the well-characterized model for human IECs, differentiated Caco-2 cells (Roxström-Lindquist et al., 2005; Ma’ayeh et al., 2017; Ma’ayeh et al., 2018; Liu et al., 2020a; Liu et al., 2020b; Peirasmaki et al., 2020), and *G. intestinalis* WB-C6 (assemblage A) trophozoites, encysting cells (7 h encystation, Uppsala encystation medium) and water-treated cysts (see Materials and Methods). The 7 h encysting time point was selected due to that *Giardia* express high levels of cyst wall proteins, the number of encystation-specific vesicles (ESVs) per cell peak and it has earlier been shown that the parasite will continue to differentiate to cysts after passing a “point of no return” after 3-6 h of encystation (Sulemana et al., 2014; Einarsson et al., 2016). It should be noted here that the population of cells in the 7 h encysting sample is heterogeneous with trophozoites and cells at various stages of early encystation, but no mature cysts will be present. The sample with water-resistant cysts also contains late encysting cells and cellular debris from lysed trophozoites and encysting cells. During infection by *Giardia*, all these stages are present at the same time but at different ratios depending on where in the intestine one looks (Campbell & Faubert, 1994; Barash et al., 2017; Pham et al., 2017). The cells in the duodenum will mainly interact with trophozoites and encysting cells whereas cells in the colon will be exposed to cysts and fragments from trophozoites and encysting cells. Thus, it is important to find out what each life cycle stage contributes with during an infection.

The different cell types interacted *in vitro* for 1.5, 3, and 4.5 h. Total RNA was extracted from the interacting cells in biological triplicates and analyzed using dual RNA sequencing (Dual RNAseq) to detect reads from both the host and parasites. Between 1x10^7^ and 4x10^7^ reads per sample were mapped to the human genome (**Figure S1**). However, it was more variable for the parasite stages: 2x10^7^ mapped reads for trophozoites, 1x10^7^ mapped reads from encysting cells, and essentially no mapped reads from cysts since very little RNA, and subsequently few reads, were generated from the hardy cysts and few were left attached to the IECs after removal of the medium (**Figure S1**). Thus, Dual RNAseq data can only be analyzed from trophozoites and 7 h encysting cells in this experiment. Clustering of the top 500 variant human genes in principal component analyses (PCA) plots showed distinct separation between the different time points from each type of interaction, but at the same time, all biological replicates clustered together (**Figure S2**). Variance explained by PC1 follows a sequential temporal path, starting at 0 h, ending at 4.5 h post-infection, for all three datasets (top leading genes of PC1 and PC2 are illustrated in **Figure S2C**).

**Figure 1:**
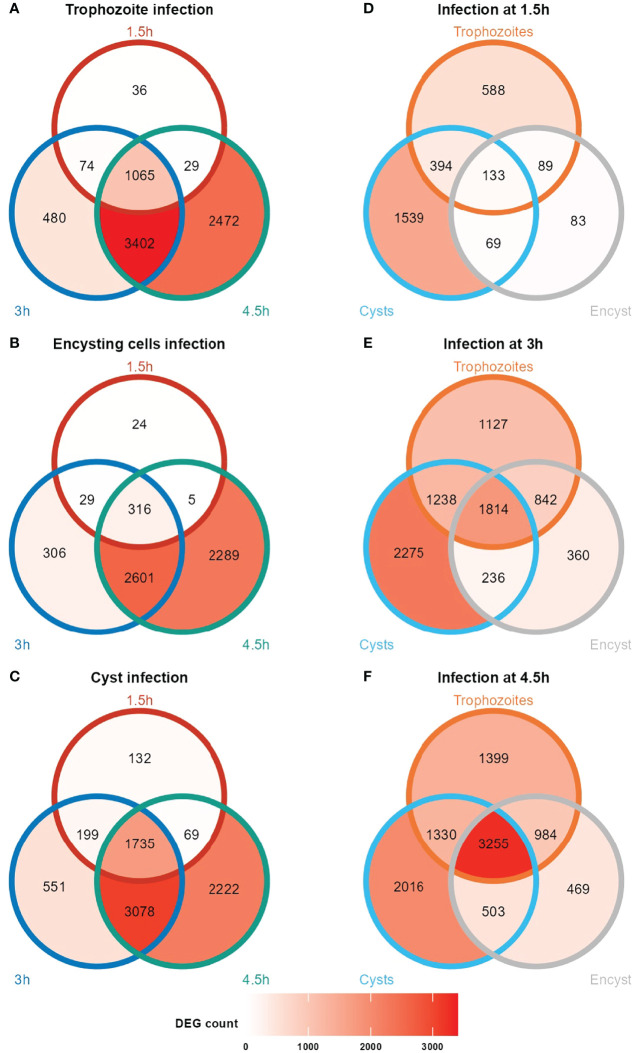
Venn diagrams showing number of differentially expressed human genes at the different time points during *Giardia* infections of differentiated Caco-2 cells. Caco-2 infection with trophozoites, encysting cells and cysts (**A**, **B**, **C**, respectively) at timepoints 1.5, 3 and 4.5 h post- infection. Unique and common DEGs at the infection time points (1.5, 3 and 4.5 h post- infection) for the different life cycle stage infections are shown in **(D–F)**. Encyst, Encysting cells.

Bioinformatic analyses (Materials and Methods) were used to identify significantly differentially expressed genes (DEGs), and the results are summarized in **Figure 1** and **Tables S1** **,** **S2**. The number of DEGs peak at the 3 h time point where most of the unique DEGs were observed (**Figures 1A–C**), and the 3 and 4.5 h timepoints are more similar than the 1.5 h timepoint (**Figures 1A–C**). Cysts induced the largest gene expression changes in the Caco-2 cells at all time points, and already after 1.5 h of interaction, there were 2135 DEGs (1326 up- and 809 down-regulated), compared to 1205 DEGs (827 up- and 378 down-regulated) in trophozoites and 374 DEGs in encysting cells (184 up- and 190 down-regulated). When DEGs were compared between the different life cycle stages (**Figures 1D–F** and **Table S2**) we identified 588, 83 and 1539 genes uniquely regulated after 1.5 h, in trophozoites, encysting cells and cysts, respectively (**Figure 1D**). The number of unique DEGs at the different timepoints increased over time (**Figures 1E, F**). Hierarchical clustering of up- or down-regulated Caco-2 DEGs of the different RNAseq data sets show a clustering of the data according to the stage of the parasite, except for the 1.5 h interaction timepoint where all 3 stages cluster together (**Figure S3**). Thus, there are clear differences between different parasite life cycle stages in their ability to induce DEGs in the interacting host cells.

### GO Term Analyses of DEGs After Host-Parasite Interactions

**Figure 2:**
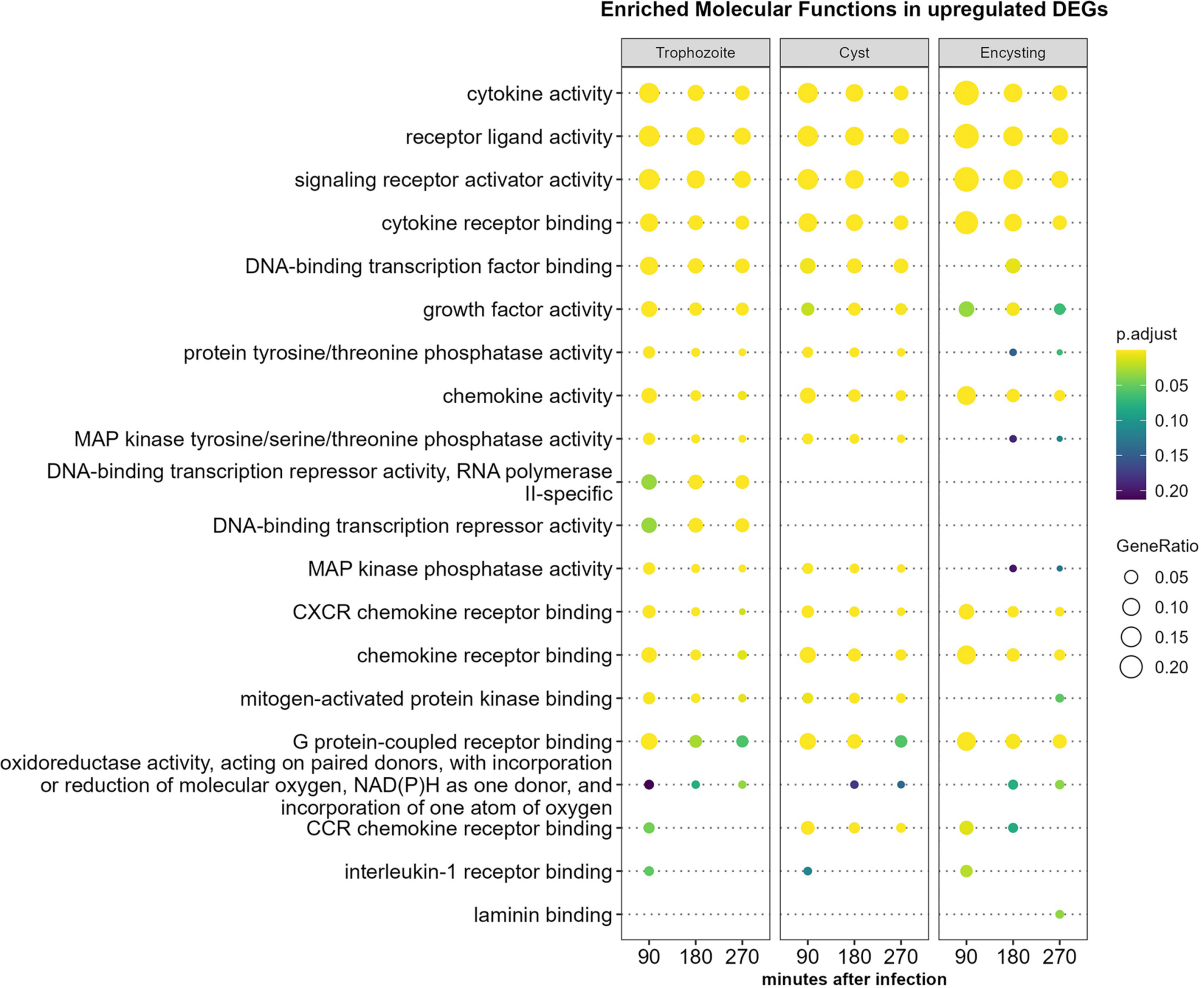
Molecular function enrichment analysis of up-regulated human genes (more than two-fold) during *Giardia* infections of differentiated Caco-2 cells. Comparison of the top 10 gene ontology (GO) terms in molecular function in each of the different *Giardia* stage infections (trophozoite, cyst and encysting cells Caco-2 infections) and infection timepoints (1.5, 3, and 4.5 h post-infection). Circle size and color indicate the gene ratio (number of differentially expressed genes against the number of genes associated with a GO term) and their significance, respectively. GO, gene ontology; MF, molecular functions.

We performed GO term-based analyses of the DEGs to understand what host pathways are affected by the different parasite stages. An analysis of up-regulated DEGs based on enriched molecular functions (**Figure 2** and **Table S3**) showed major enrichments of genes involved in chemokine and cytokine signaling after interactions with parasites from all stages. There is also enrichment for genes involved in MAP kinase pathways, but it is less pronounced in encysting cells (**Figure 2**). Trophozoites show enrichment of many transcriptional repressors (ZBTB20, HEY1, BTG2, IRF8, **Table S3**), and this was not seen after interaction with cysts of encysting cells (**Figure 2**). We could verify most of these observations using analyses of enriched Reactome pathways (**Figure S4** and **Table S3**). However, we could also increase the resolution of the analysis and identify specific pathways that are affected by parasite interactions. Trophozoites and encysting cells up-regulate genes involved in cholesterol biosynthesis and genes involved in cholesterol regulated gene expression *via* SREBP and SREBF, but this is not seen to the same degree during cyst interaction (**Figure S4**). One example is NSIG1, which blocks the processing and activation of SREBP (**Table S3**). Parasite-induced effects on the hosts’ cholesterol metabolism have also recently been seen in human enteroids interacting with *Giardia* WB trophozoites (Holthaus et al., 2021). *Giardia* is dependent on cholesterol scavenging from the host during active growth (Stevens et al., 1997; De Chatterjee et al., 2015; Mendez et al., 2015). Our data suggest that this affects gene expression in the IECs in a life cycle stage-specific manner.

Cysts induce up-regulation of tropomyosin related kinase A (TRKA) signaling, which is not seen after trophozoite or encysting cell interaction (**Figure S4**). TRKA is expressed in the small intestine, and it signals from either the plasma membrane or endosomes to affect the proliferation and differentiation of neural cells (Esteban et al., 1995). Nerve growth factor (NGF)-stimulated transcription is also specifically stimulated by cysts (**Figure S4**), and it can bind and signal *via* TRKA. In addition, G protein-coupled receptors (GPCRs) are enriched for when cysts and encysting cells interact with host cells (**Figure S4**), showing differences in extracellular signaling in the IECs induced by the different life cycle stages of *Giardia*.

Cysts show a stronger induction of the Toll-like receptor 3 (TLR3) cascade and MyD88-independent and TRIF/TICAM1 TLR4 pathways (**Figure S4** and **Table S3**). The MyD88-independent signaling pathway is shared by the pattern recognition receptors (PRRs) TLR3 and 4, and both PRRs are expressed in the human duodenum (Eiró et al., 2012). TRIF is a key adapter-molecule transducing signals from activated TLR3 and 4 receptors in a MyD88 independent fashion. It is up-regulated by all stages of *Giardia* but highly already after 1.5 h of interaction with cysts (**Table S1**). TRIF interacts with TRAF3 and TRAF6, and these adapter proteins are also up-regulated in Caco-2 cells upon *Giardia* interactions (**Table S1** **,** **S3**). Signaling from TLR3 and 4 results in NFkappaB and IRF induction and cytokine/chemokine induction. It is possible that cysts have surface exposed proteins/sugars that induce specific cytokines/chemokines (see below).

**Figure 3:**
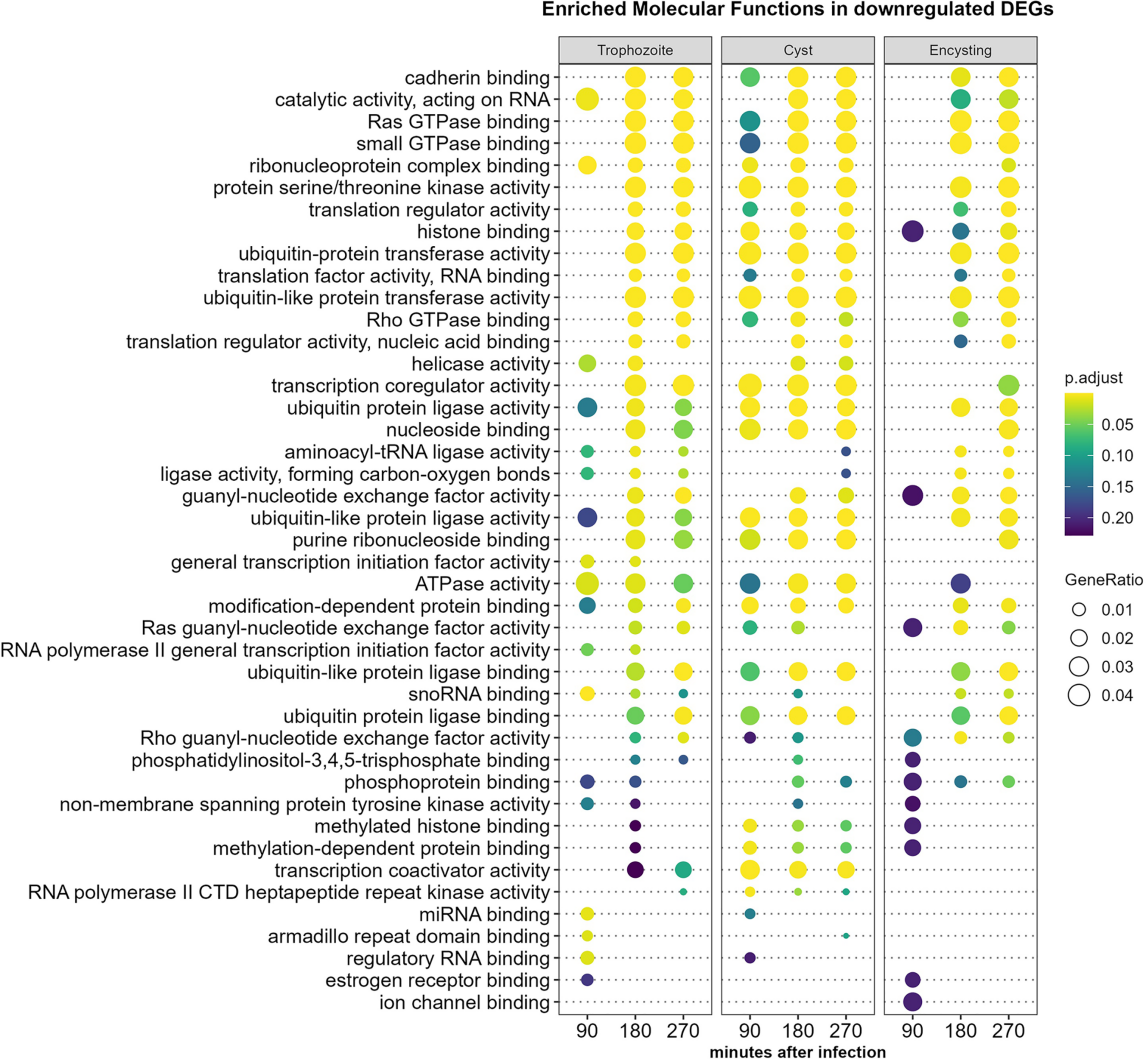
Molecular function enrichment analysis of down-regulated Caco-2 genes (more than twofold) during *Giardia* infections. Comparison of the top 10 gene ontology (GO) terms in molecular function in each of the different *Giardia* stage infections (trophozoite, cyst and encysting cells Caco-2 infections) and infection time points (1.5h, 3h, and 4.5h post-infection). Circle size and color indicate the gene ratio (number of differentially expressed genes against the number of genes associated with a GO term) and their significance, respectively. GO, gene ontology; MF, molecular functions.

A GO term analysis using molecular function enrichment of down-regulated genes during *Giardia*-host cell interactions shows larger overall differences than when up-regulation is studied (**Figure 3**). The major groups of down-regulated functions are similar between the parasite stages. However, the response is faster in the cyst interaction, especially in the proteasome/ubiquitin pathways, transcription coregulator, and activator activity histone binding (**Figure 3**). Exceptions are miRNA binding, regulatory RNA binding, and general transcription activation activity, which are faster down-regulated in trophozoites, and tRNA aminoacylation, which is quickly down-regulated in trophozoites and encysting cells (**Figure 3**). Again, Reactome enrichment shows larger resolution, and cysts show fast down-regulation of degradation pathways of DVL and AXIN, which are both involved in the regulation of Wnt signaling (**Figure S5**) (Kurokawa et al., 2020). There are also larger effects on chromatin organization and modification and Dectin-1 mediated noncanonical NF-kappa B signaling by cysts (**Figure S5**). Dectin-1 is a C-type lectin, and it is a PRR involved in recognizing beta 1,3 and 1,6-linked glucans (Kalia et al., 2021). In addition, Dectin-1 has recently been shown to bind the *Toxoplasma gondii* oocyst surface (Fabian et al., 2021) but it is not known if it recognizes the sugars in the *Giardia* cyst wall.

### DEGs in differentiated Caco-2 Cells After *G. intestinalis* Trophozoite Interactions

**Figure 4:**
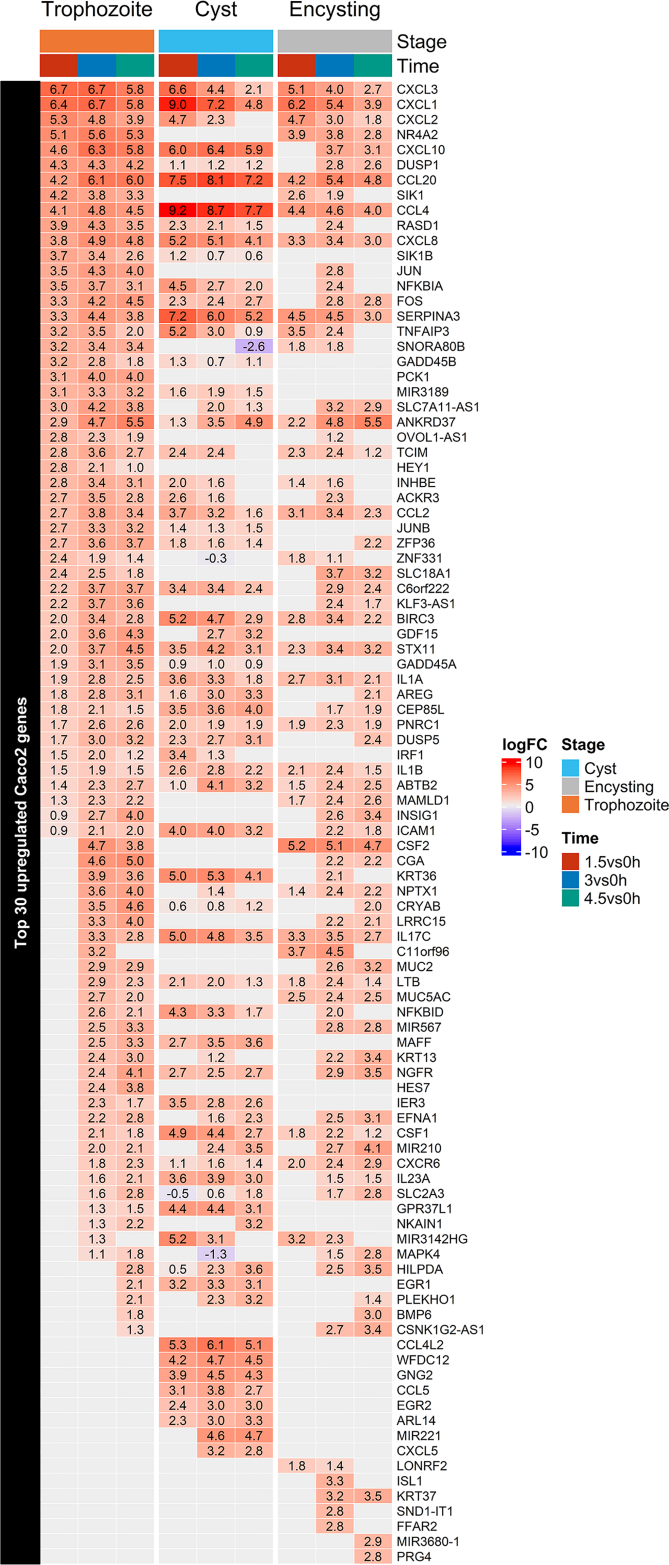
Heatmap of the top 30 up-regulated Caco-2 genes during *Giardia* trophozoite, cyst and encysting cells infections in each of the infection timepoints, 1.5, 3, and 4.5h. Only significant values are shown, insignificant values are set 0.

**Figure 5:**
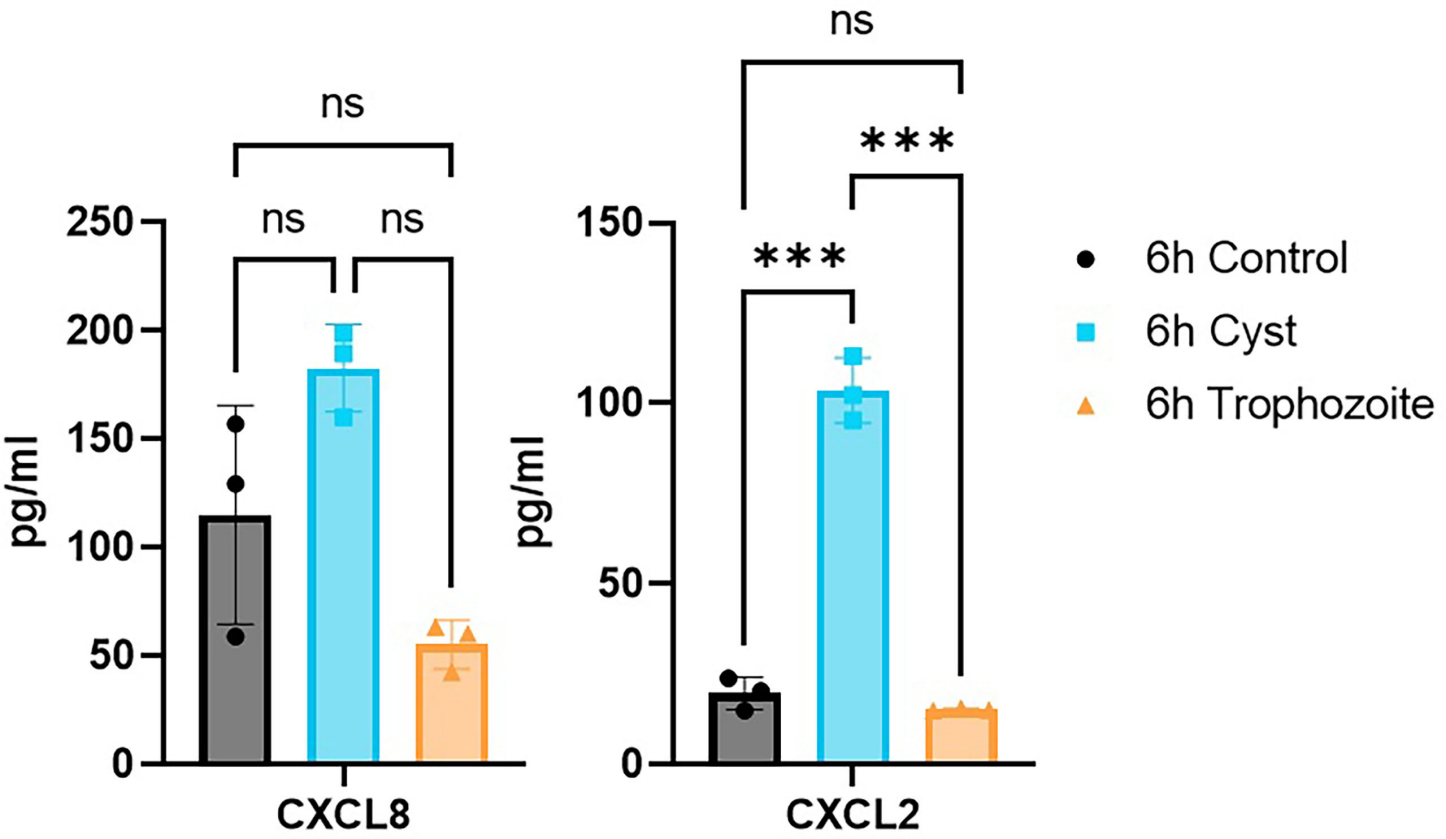
ELISA measurements of chemokines C-X-C chemokine ligand 8 (CXCL8) and C-X-C chemokine ligand 2 (CXCL2) in the culture media after 6h of Caco-2 infections with *Giardia* trophozoites and cysts. ***P < 0.001; ns, non-significant.

Among the 6968 DEGs identified in Caco-2 cells after trophozoite interactions for 4.5 h, 1399 are unique for trophozoites (**Figure 1**; **Tables S1** **,** **S2**). The 30 most highly up-regulated genes in Caco-2 cells after trophozoite interactions over all three time points are presented in **Figure 4**. In line with earlier expression studies, genes involved in innate immune responses, regulation of the transcription factors NF-kappaB and AP-1, MAPK signaling, mRNA decay regulation, regulation of cell proliferation and apoptosis, and glucose metabolism are enriched in the top up-regulated genes (**Figure 4**) (Roxström-Lindquist et al., 2005; Ma’ayeh et al., 2018). The chemokines are the most highly up-regulated group of genes, and they are already highly up-regulated after 1.5 h of interaction (**Figure 4**). RT-PCR analyses of selected chemokines (CXCL1 to 3, CXCL8 or IL8 and CCL20) showed that up-regulation of the transcript levels can be seen already after 15 and 30 min of interaction between trophozoites and Caco-2 cells (**Figure S6**). **Figure 4** also shows the top 30 up-regulated genes after interaction with encysting cells and cysts. The most up-regulated chemokines after trophozoite interactions (CXCL1 to 3, CXCL10, CCL20, CCL4, CXCL8, CCL2, IL17C, IL23A, IL1A, and IL1B) are also up-regulated by encysting cells and cysts (**Figure 4**). However, the level and timing of up-regulation differ (**Figure 4**). A faster and higher level of up-regulation of CXCL1, CCL20, CCL4, IL23A is seen after cyst interactions (**Figure 4**), and three chemokines are only up-regulated during the cyst interaction (CCL4L2, CCL5, and CXCL5, **Figure 4**). The level of up-regulation of selected chemokines was also studied using ELISA, and this showed that CCL4 and CCL5 were below the detection level, but CXCL2 and CXCL8 could be detected. It is clear that the proteins levels are lower after trophozoite incubation (**Figure 5**). This can be due to an active down-regulation of expressed chemokines by released proteases from trophozoites, as shown in earlier studies (Ma’ayeh et al., 2017; Liu et al., 2018), but this mechanism seems to be less active in the cyst preparation (**Figure 5**).

The immediate early gene ZFP36 or TTP has earlier been shown to be up-regulated by excretory-secretory products (ESPs) from *Giardia* (Ma’ayeh et al., 2017). It is an anti-inflammatory protein that regulates the stability of many chemokines, including all chemokines in the top 30 up-regulated genes after trophozoite interactions (**Figure 4**) (Kovarik et al., 2021). Western blot analyses show that TTP is up-regulated on the protein level during *Giardia* trophozoite-host cell interactions (**Figure S7**). This is in contrast to Regnase-1 or ZC3H12A, a ribonuclease regulating inflammatory signaling, which is rather up-regulated early during the interaction (**Figure S7**).

Mucins play an important role as a protective barrier against infectious diseases in the intestinal regions (Johansson & Hansson, 2016). The gel-forming mucins Muc2 and Muc5AC are up-regulated by trophozoites but not cysts (**Figure 4**). Muc2 is the main gel-forming mucin in the intestinal region, and it has been shown to play an important role during *Giardia* infections (Amat et al., 2017). Cysteine protease activities from *Giardia* trophozoites degrade Muc2, and there is a compensatory up-regulation of Muc2 expression (Amat et al., 2017). Muc5AC is usually expressed in the stomach, but it can increase expression in the intestine during infections (Johansson & Hansson, 2016). There is significant expression variability among the surface-bound mucins: Muc13 and Muc17 are more up-regulated by cysts, Muc3A and Muc12 are up-regulated by trophozoites and encysting cells. Muc4 is down-regulated by trophozoites while Muc20 is down-regulated by trophozoites and cysts (**Figure 5** and **Table S1**). Thus, it is clear that there is a differential effect of different *Giardia* life cycle stages on mucin expression in intestinal cells.

The peptide hormone cholecystokinin (CCK) is induced during *Giardia* infections resulting in smooth muscle contractions mediated *via* mast cells (Li et al., 2007). The CCK hormone has also been associated with post-giardiasis symptoms (Dizdar et al., 2010). Here we can see that expression of CCK is highly up-regulated in the IECs after 4.5 h contact with trophozoites (**Table S1**), but this is not seen with the other life cycle stages. Another hormone specifically up-regulated by trophozoites and encysting cells is angiopoietin-like protein 8 (C19orf80), which regulates serum triglyceride levels (**Table S1**).

### DEGs in Differentiated Caco-2 Cells After Interactions With Encysting Cells and Cysts

7104 genes are differentially expressed after the interaction of cysts with Caco-2 cells for 4.5 h, and 2222 are unique DEGs for cysts (**Table S1****,** **S2**). Many transcription factors can be found among the DEGs; AP-1, NR4A2 is more highly induced by trophozoites than cysts, and NF-kappa B inhibitors are more highly up-regulated by cysts (**Figure 4** and **Table S1**). The early growth response (EGR) has earlier been shown to be activated by assemblage B trophozoites (Ma’ayeh et al., 2018), and here it is more highly activated by cysts than trophozoites (EGR1, 2 and 4, **Figure 4** and **Table S1**).

Immunomodulatory molecules change their expression during *Giardia* cyst interactions. The chemokine profile is different compared to trophozoites, the main up-regulated chemokines are the same, but CCL4, CCL4L2, CSF1, CCL5, CXCL5, and CX3CL1 are more highly up-regulated in the cyst interaction (**Figure 4** and **Table S1**). RT-PCR analyses verified expression differences of the chemokine CCL20 dependent on (**Figure S8**). Serpin A3 is a serine peptidase inhibitor that is more highly up-regulated by cysts (**Figure 4**), and it can inhibit neutrophil cathepsin G and mast cell chymase (Baker, 2007). WFDC12 is an anti-bacterial protein and a putative serine peptidase inhibitor, and it is also up-regulated only during cyst interactions (**Figure 4**). These observations, together with differences in chemokine expression profiles and a higher up-regulation of TLR3 and TLR4 regulatory pathways, suggest that cysts (and late encysting cells) can induce unique innate immune responses during *Giardia* infections.

A large number of genes (1074) are specifically down-regulated in Caco-2 cells after cyst interactions (**Table S1** **,** **S2**). The negative regulator of the innate immune response, NLRC3, is down-regulated by cysts, stimulating TLR4 signaling *via* TRAF6 (Li et al., 2020). Among the most down-regulated genes during cyst-Caco-2 interactions, we also find many histone encoding genes and long non-encoding RNAs (**Table S1**).

### DEGs in Encysting *Giardia* Cells During Interaction With Differentiated Caco-2 Cells

The most abundant DEGs in trophozoites interacting with differentiated Caco-2 cells have recently been shown to encode hypothetical proteins and members of the High Cysteine Membrane Protein (HCMP) family (Peirasmaki et al., 2020). Among the highly up-regulated genes there are also proteins involved in proteolysis, cellular redox balance, as well as lipid and nucleic acid metabolic pathways (Peirasmaki et al., 2020). A major difference in this trophozoite interaction experiment compared to earlier studies was a 2 h pre-incubation of the trophozoites in DMEM before addition to the Caco-2 cells to reduce the medium effects have been seen to dominate gene expression changes in earlier experiments (Ringqvist et al., 2011) and in the experiment presented here, we also used a 2 h preincubation in DMEM.

**Figure 6:**
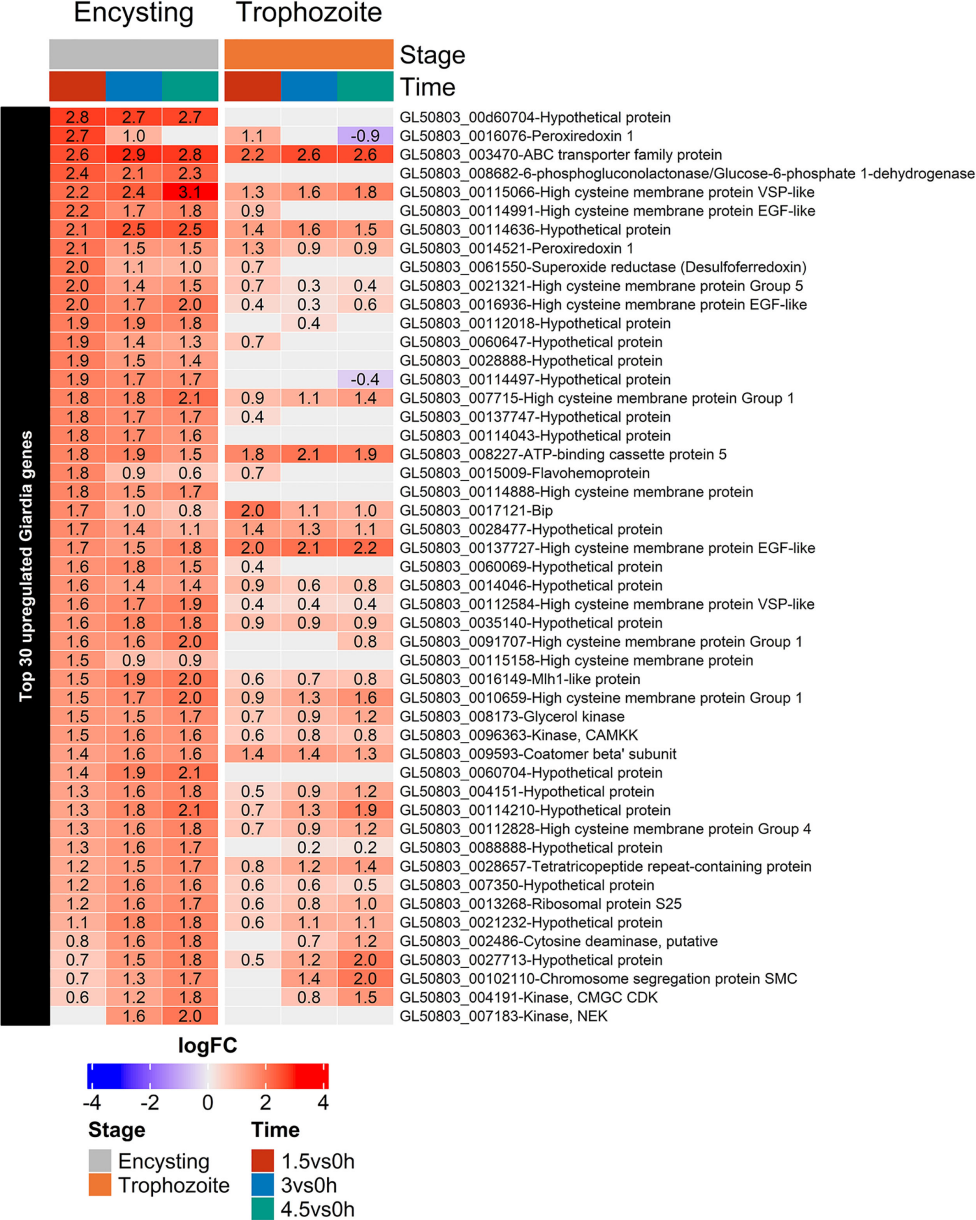
Heatmap of the top 30 up-regulated genes in encysting *Giardia* cells during Caco-2 infections at timepoints 1.5, 3, and 4.5h during Caco-2 infection compared to trophozoite differentially regulated genes at the same infection timepoints. Only significant values are shown, insignificant values are set 0.

*Giardia* quickly adapts to the interaction with the human cells: 160 *Giardia* genes are up-regulated and 138 genes are down-regulated more than 2-fold after 1.5 h interaction between 7 h encysting cells and differentiated Caco-2 cells (**Table S4**). After 4.5 h 253 genes are up-regulated and 260 genes are down-regulated more than 2-fold. Among the 30 most up-regulated genes at all timepoints in the 7 h encysting cells (**Figure 6**), 11 are also found in an earlier microarray data set from trophozoite-Caco-2 cell interaction (ORFs 7183, 7715, 8173, 8682, 10659, 16936, 91707, 112584, 114210, 115066, and 137727), and 7 are HCMPs (Ringqvist et al., 2011). If the top 30 up-regulated genes in 7 h encysting cells are compared to the trophozoites interacting with Caco-2 cells and analyzed by RNAseq (Peirasmaki et al., 2020), there are 9 overlapping genes (ORFs 102110, 4191, 137727, 27713, 10659, 114210, 114636, 3470, and 115066) and 2 are HCMPs. Only 4 of the 30 top up-regulated genes overlap between the three different experiments; 3 HCMPs (ORFs 10659, 115066, and 137727) and one hypothetical surface protein (ORF 114210). There are 14 genes among the top 30 most up-regulated genes that are only found using 7 h encysting cells; 8 are hypothetical proteins and 2 HCMPs (**Figure 6** and **Table S4**).

An analysis of a larger number of up-regulated genes shows that the encysting cells up-regulate several proteins involved in protein transport (ORFs 9593 Coatomer beta, 10803 Qa-SNARE 5, 4192 ARF-like protein 2b and 5161 Qb-SNARE 3, **Table S4**). Oxidative stress is clearly a factor already after 1.5 h of the interaction with 7 h encysting cells with up-regulation of ORFs 14521 and 16076 Peroxiredoxin 1, 61550 Superoxide reductase, 15009 Flavohemoprotein, 4946 Peptide Methionine Sulfoxide Reductase, msrA, 17150 NADPH oxidoreductase, 6289 FixW protein, and 9355 Thioredoxin-like protein (**Table S4**). This is followed at 3 and 4.5 h by an increase in DNA repair with ORFs 16149 Mlh1-like protein, 2486 Cytosine deaminase, 102110 SMC protein, and 7718 DDI1-like DNA damage inducible protein (**Table S4**).

**Figure 7:**
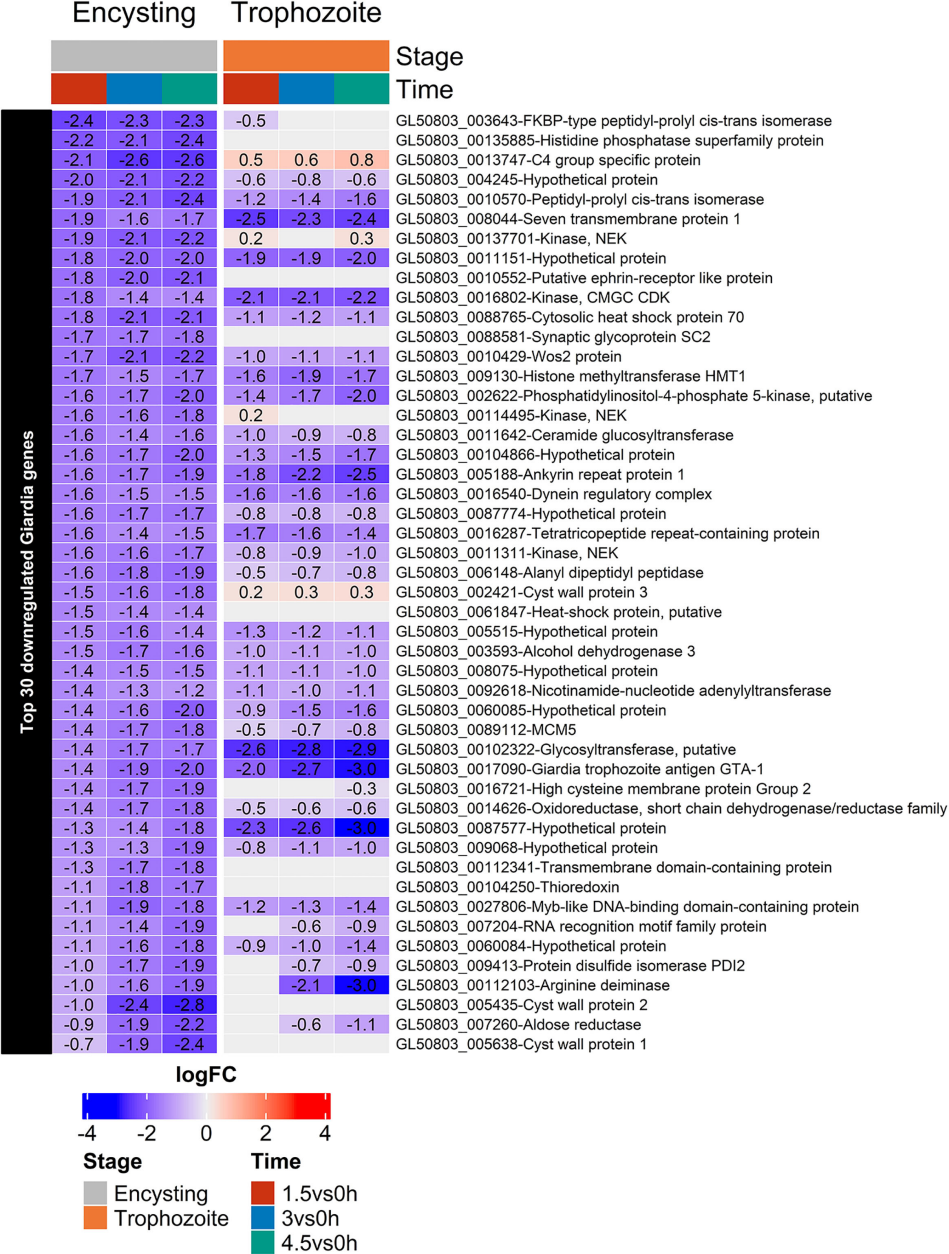
Heatmap of the top 30 down-regulated genes in encysting *Giardia* cells during Caco-2 infections at timepoints 1.5, 3, and 4.5h during Caco-2 infection compared to trophozoite differentially regulated genes at the same infection timepoints. Only significant values are shown, insignificant values are set 0.

Genes encoding kinases, regulators of the cell cycle, and arginine metabolism and cytoskeletal proteins were the main down-regulated genes in the interacting trophozoites (Peirasmaki et al., 2020) (**Figure 7**). This can also be seen in the encysting cells (**Figure 7** and **Table S4**), but the most down-regulated genes in 7 h encysting cells during host cell interactions are up-regulated genes during encystation (Rojas-López et al., 2021); ORFs 5638, 5435, and 2421 cyst wall proteins 1 to 3, 4245 and 11151 hypothetical proteins, 114495 and 137701 NEK kinases, 10552 Ephrin-like receptor, 14626 Oxidoreductase, 112341 Transmembrane protein, 27806 Myb-like protein, 113610 GlcNAc-PI synthesis, and 88581 synaptic glycoprotein (**Table S4**). This suggests that encysting cells are inhibited in the encystation process during the interaction with Caco-2 cells, and follow-up experiments showed that fewer cysts are produced when 7 h encysting cells are transferred to DMEM or to interacting Caco-2 cells (**Figure S9**). Many stress-related genes are also down-regulated in the 7 h encysting cells during Caco-2 cell interactions (**Figure 7**, ORFs 13747, C4 stress-related protein, 88765 cytoplasmic Hsp70, 3643 and 10570 peptidyl-prolyl cis-trans isomerase, and 9413 protein disulfide isomerase-2).

To conclude the parasite responses, differential responses are induced in trophozoites and encysting cells during interaction with intestinal cells, and the small set of overlapping genes are mainly HCMPs. Furthermore, the encystation process is affected by the presence of intestinal cells.

## Discussion

In this study, we have used an *in vitro* system for studies of *Giardia*-host cell interactions to identify differentially expressed genes in the parasite and host cells: differentiated Caco-2 cells as host cells and *G. intestinalis* WB parasites from different stages of the life-cycle (trophozoites, early encysting cells, and cysts). In the host cells, gene expression changes were mainly found in genes involved in immune and MAPK signaling, apoptosis and oxidative stress responses.

Humans are infected by *G. intestinalis* parasites from two main genotypes or assemblages (Assemblage A and B) and genomic differences between assemblage A and B parasites (80% identity on nucleotide level) have suggested that this is actually two different species (Franzén et al., 2009). Previously, the transcriptome of Caco-2 cells infected with *Giardia* trophozoites of the isolate GS (assemblage B) was measured using the same infection set-up as in this study (Ma’ayeh et al., 2018). GS infected differentiated Caco-2 cells shared 44.5% (98 genes), 69.3% (941 genes), and 70.2% (1756 genes) of the DEGs induced by the WB trophozoites (assemblage A) at the 3 time points (1.5, 3, and 4.5 h). However, even though the detected DEGs differ between GS and WB infected Caco-2 cells, the GO term enrichment analysis showed a core of similar GO term enrichments. It is clear that apoptosis (Liu et al., 2020a; Liu et al., 2020b), oxidative and metabolic stress is induced by *Giardia* trophozoites from both assemblages. Molecular function enrichment analysis showed that 6.53% of up-regulated DEGs at 1.5 h were involved in transcription factor binding (GO:0008134), which decreased to 1.93 and 1.44% at time points 3 and 4.5 h, respectively. Caco-2 cell infection with GS trophozoites showed that the important transcription factors nuclear factor kappa B (NFKB) and activating protein-1 (AP-1) were enriched for, as well as mitogen-activated protein kinase (MAPK) signaling pathway genes (Ma’ayeh et al., 2018). All three observations could be confirmed during WB trophozoite infection in this study. The differences in DEGs in the Caco-2 cells are actually larger between different life cycle stages of the WB isolate than the differences in DEGs identified between GS and WB trophozoites.

Immune response genes are the most up-regulated genes during the *Giardia’s* interaction with differentiated Caco-2 cells, no matter if it is assemblage A or B or different life cycle stages. Differentially transcribed immune response genes are collected in **Tables S1** **,** **S3**, and already after 1.5 h interaction, the level of up-regulation is high. Our RT-PCR data show that up-regulation starts after 15 min of interaction, peaking around 3 to 4.5 h (**Figure S6**). At all time-points with all life-cycle stages, the most up-regulated genes are a set of chemokines (CXCL -1, -2, -3, -8, 10, and CCL-2, -4 and -20, **Table S1**). However, there are differences in the level of up-regulation between the different WB life cycle stages and with assemblage B trophozoites (Ma’ayeh et al., 2018). CSF2 is up-regulated by WB trophozoites and encysting cells but not cysts, and the chemokines CCL4L2, CCL5, and CXCL5 are only up-regulated by cysts (**Figure 4**). IL23A and TNFSF9 were not detected in the GS trophozoite interaction experiments (Ma’ayeh et al., 2018) and they are both more highly up-regulated by cysts (**Figure 4**). Several cytokine receptors are differentially expressed during the *Giardia* infections, including IL11RA, IFNGR1, IFNGR2, IL10RA, and ACKR3. The atypical chemokine receptor 3 (ACKR3) or CXCR7 is a chemokine-scavenging receptor, which is highly up-regulated during WB trophozoite infections at all 3 time points, show a much lower level of up-regulation by encysting cells and cysts (**Figure 4**). In contrast to WB trophozoite infection, ACKR3 is only differentially transcribed at 1.5 h during GS infection but not later. The exact function of ACKR3 during immune responses is still poorly understood. However, it is known that it acts as a decoy receptor for CXCL12 and CXCL11, and it also binds human herpesvirus 8-encoded chemokine vCCL2/vMIP-II (Szpakowska et al., 2016). ACKR3 expression has previously been shown to be up-regulated during inflammation by IL-8, IL-1 beta, LPS, and during oncovirus infections (Quinn et al., 2018).

*Giardia* infections of IECs quickly up-regulate chemokines, but the expression decreases after 3 to 4.5 h (**Figure S6**). We looked for chemokine and immune response regulators that could explain this regulation. We found that Tristetraprolin (TTP), Nocturnin (NOCT or CCR4NL), and Zinc Finger CCCH-Type Containing 12A (ZC3H12A or Regnase-1) were all up-regulated during the infection (**Table S1**). TTP, also known as zinc finger protein 36 homolog (ZFP36), has been implicated with important functions in inflammation regulation, cancer development, and recently also in infection (Kovarik et al., 2021). Transcription of TTP was up-regulated at all time points (6.3, 11.9, and 12.9-fold at 1.5, 3, and 4.5 h, respectively) during WB trophozoite IECs infections. Up-regulation is also seen with cysts but to a lower level (3.5, 3.0, and 2.6-fold, **Figure 4**). TTP can mediate mRNA decay by binding to AU-rich elements (AREs) in the 3’UTRs and introns of mRNAs of inflammatory, signaling cascades, and apoptosis genes (Kovarik et al., 2021). Influenza A virus (IVA) infection has been shown to induce TTP *via* the RIG-I pathway (Dudek et al., 2016). Interestingly, *Giardia* infection up-regulates several downstream RIG-I signaling pathway genes during the infection (**Table S2**), such as TRAF3, IKK, and TBK1. Furthermore, pathogenic bacteria can also manipulate TTP levels *via* the bacterial secondary messenger cyclic-di-AMP (c-di-AMP) (Mahmoud et al., 2020). Thus, up-regulation of TTP might be a general immunosuppressive mechanism used by pathogenic microbes to suppress an initial up-regulation of inflammatory mediators and this can be studied further in the *Giardia*-Caco-2 system.

Studies of giardiasis patients have shown an increased number of apoptotic cells in the duodenum (Troeger et al., 2007). Recent studies of *Giardia*-host cell interactions using 3D stem cell-enriched organoid cultures from human duodenal biopsies and WB trophozoites show induction of apoptosis after 48 h interaction (Holthaus et al., 2021). WB parasites of all life-cycle stages up-regulate pro-apoptotic and anti-apoptotic genes (**Table S1**). This ambiguity was first observed when Giardia WB trophozoite-Caco-2 interactions were studied using microarrays (Roxström-Lindquist et al., 2005), and it was also observed during GS trophozoite infection of Caco-2 cells (Ma’ayeh et al., 2018). Differentially transcribed apoptosis-related genes are listed in **Figure S4**. Both intrinsic and extrinsic apoptosis pathway genes were identified as DEGs during infection. GO term enrichment analysis showed that the regulation of apoptotic process (GO:0042981) was enriched in up-regulated DEGs during all 3 time-points from all three life cycle stages. Extrinsic apoptosis pathway genes were differentially expressed (e.g., TNFRSF10A, B, and D) and GO term enrichment showed that tumor necrosis factor-mediated signaling pathway (GO:0033209) and negative regulation of extrinsic apoptotic signaling pathway (GO:2001237) were enriched for in the DEGs. This is in line with a recent report (Liu et al., 2020b) showing that *Giardia* trophozoites can activate CASP3/8 signaling pathways *via* activation of TNFR1 and K63 de-ubiquitination of RIP1, which in turn is caused by up-regulation of CYLD and A20. GO term enrichment analysis also detected the term intrinsic apoptotic signaling pathway in response to endoplasmic reticulum stress (GO:0070059). CASP9 is a central player in the intrinsic apoptosis pathway, and both pro-and anti-apoptotic genes influencing its activation are differentially transcribed during infection. CASP9 can activate CASP3, which activates apoptosis in the cell in return. CASP9 was differentially up-regulated after 3 and 4.5 h post infection (2.2 and 1.9-fold up-regulation, respectively). Anti-apoptotic genes in the intrinsic apoptosis pathway were mainly up-regulated after 3 or 4.5 h and included BCL2L1, MCL1, and PIM3. Pro-apoptotic DEGs such as DIABLO, BBC3, APAF1, BNIP1, and PMAIP1 were up-regulated after 1.5 h (PMAIP1, BNIP1), 3 h (DIABLO, BBC3), and after 4.5 h (APAF1), and all pro-apoptotic DEGs stayed up-regulated after the initial up-regulation. It was also recently shown that *Giardia* WB trophozoites induce Caco-2 cell apoptosis through a reactive oxygen species (ROS) and mitochondria-mediated caspase-dependent pathway (Liu et al., 2020a).

A highly up-regulated gene during *Giardia* infections of Caco-2 cells is C6orf222 (**Figure 4**). C6orf222 is up-regulated during all 3 time-points with WB trophozoites (4.7, 12.8, and 12.7-fold up-regulation at 1.5, 3, and 4.5 h, respectively), which is slightly higher than in interactions with cysts and encysting cells (**Figure 4**). It is also highly up-regulated during GS trophozoite infections [30.1, 18.3, and 7.6-fold up-regulation at 1.5, 3, and 4.5 h, respectively, (Ma’ayeh et al., 2018)]. C6orf222 has been shown to interact with known apoptosis regulators and contains a BH3 motif. A study that identified binding partners of pro-survival Bcl-2 proteins detected that C6orf222 is binding to 5 pro-survival proteins Bcl-X(L) (isoform of BCL2-L1), Bcl-w (BCL2L2), Bcl-2, Mcl-1, and Bfl-1 (BCL2A1) (DeBartolo et al., 2014). Furthermore, the human reference protein interactome mapping project (HuRI) showed that C6orf222 interacts with BCL2L1, BCL2L2, BNIP2, and MAPK9 (Luck et al., 2020). The interaction data thus suggests a functional role in apoptosis during infections for C6orf222, and further studies of this poorly characterized human ORF and its putative role in apoptosis can be performed in the *Giardia*-Caco-2 system.

To summarize, it is clear that apoptosis is an important factor during *Giardia’s* interaction with host cells, but the responses are complex, and further experiments are needed *in vivo* and in non-transformed model systems like enteroids (Holthaus et al., 2021) in order to define the role of apoptosis during giardiasis.

The production of RO and nitric oxide (NO) are innate defense mechanisms used by the human cells against *Giardia* and other intestinal pathogens (Ringqvist et al., 2011; Stadelmann et al., 2012; Ma’ayeh et al., 2015). From the expression data here it is clear that *Giardia* up-regulate many genes involved in oxidative stress regulation but the produced ROS and NO are not only harmful against *Giardia* trophozoites but all types of cells. Therefore, the human host cells also need to up-regulate antioxidant proteins during *Giardia* interaction (Stadelmann et al., 2012; Stadelmann et al., 2013; Ma’ayeh et al., 2018). GO terms corresponding to antioxidative responses and hypoxia are induced in the Caco-2 cells during parasite interactions (Ma’ayeh et al., 2018), and this was also recently seen during interactions with enteroids (Holthaus et al., 2021). In our study, all life cycle stages induce oxidative stress responses in the Caco-2 cells, and cysts show the strongest and fastest up-regulation (**Table S1**). Nuclear factor, erythroid 2-like 2, (NFE2L2/NRF2) is the master regulator of intracellular antioxidant responses (Jaiswal, 2004), and this transcription factor is up-regulated by cysts already after 1.5 h of *Giardia* interaction. It stays up-regulated to 4.5 h (**Table S1**). NFE2L2 regulates the transcription of genes containing antioxidant response elements (ARE) in their promoters, thereby inducing the expression of multiple antioxidant proteins (Itoh et al., 1997). Several of the NFE2L2-induced antioxidant genes are up-regulated after 1.5 h of cysts and trophozoite infection, such as SOD2, SRXN1, SESN2, NQO1, and GPX2 (**Table S1**). Genes involved in glutathione (GSH) synthesis and regulation are induced by NFE2L2, and these genes are important factors during the oxidative stress response. Glutathione peroxidase 2 (GPX2) belongs to the family of glutathione peroxidases, which protects the cell against oxidative damage by reducing H_2_O_2_ and hydroperoxides by glutathione (Brigelius-Flohé & Kipp, 2012). GPX2 is up-regulated both at 3 and 4.5 h post trophozoite infection (3.1 and 4.4-fold change, respectively). Another NFE2L2 regulated gene is the solute carrier family 7 member 11 (SLC7A11), which plays an important role in intracellular glutathione (GSH) synthesis and in maintaining the redox balance in the cell (Tu et al., 2021). SLC7A11 has antioxidant functions, and it is up-regulated 3 and 4.5 h (3.2, and 3.4-fold expression, respectively) post-trophozoite infection. SLC7A11 is a member of the anionic amino acid transport system that is specific for cysteine and glutamate, controlling the exchange of intracellular glutamate for extracellular cysteine (Tu et al., 2021). It is also involved in the regulation of cell death *via* apoptosis and ferroptosis (Tu et al., 2021). Interestingly its neighboring non-coding antisense RNA gene SLC7A11-AS1 is highly up-regulated by trophozoites during all time points (7.7, 17.3, and 13.2-fold up-regulated at 1.5, 3, and 4.5 h, respectively) but less by cysts (**Figure 4**). In pancreatic cancer cells, up-regulated SLC7A11-AS1 levels facilitate the stabilization of NFE2L2 and thereby reduce intracellular ROS (Yang et al., 2020). In gastric cancer, a decreased expression of SLC7A11-AS1 was correlated with higher cancer cell proliferation (Luo et al., 2017). The same study showed that a SLC7A11-AS1 knockdown resulted in up-regulation of SLC7A11, suggesting that SLC7A11-AS1 also regulates SLC7A11 expression levels. Our study shows that the intestinal protozoan parasites *Giardia* can up-regulate SLC7A11-AS1, but it is uncertain what the effect is. One alternative is that it stabilizes NFE2L2 and reduces the ROS effects in the IECs. Another alternative is that it reduces the protein levels of SLCA11. *Giardia* trophozoites consume large amounts of cysteine (Lujan & Nash, 1994), and this, together with a reduced SLC7A11 level, will result in reduced GSH levels in the IECs, higher ROS levels, lipid peroxidation, and ferroptosis induction. Further experiments will show what role the up-regulation of the lncRNA SLC7A11-AS1 plays during *Giardia* infections.

During host cell interactions the parasites mainly up-regulate hypothetical proteins, members of the cysteine-rich membrane proteins HCMPs, and, like in the host cells, oxidative stress response proteins (**Figures 6** and **7**). Many hypothetical proteins are induced in the parasites during host cell interactions and there are differences between trophozoites and encysting cells (**Figure 6**). The most highly induced hypothetical gene in encysting cells is ORF 00d60704 (**Figure 6**). This gene was identified in the new assembly of the *Giardia* WB genome (Xu et al., 2020a). It is a duplicated gene with two almost identical copies on chromosome 4, which is very uncommon in *Giardia*, and it is only found in the new version of the WB genome, as a shorter version in assemblage B (hypothetical protein QR46_4930) and in *Giardia muris*. It has no identifiable domains and modelling of the structure of this 837aa long protein, with two almost identical copies up-regulated in encysting cells but not trophozoites, gave no idea of the putative function. There are several more hypothetical proteins that are highly up-regulated in 7 hrs. encysting cells but not in trophozoites upon host cell interactions (ORFs 28888, 60647, 112018, 114043 114497 and 137747). This shows that there encysting cells respond differently to host cell contact and it will be interesting to try to identify the function of these genes.

The strongest host responses after 1.5 and 3 hrs interaction was up-regulation of different chemokines (**Figure 4**). Encysting cells and cysts can induce the same type of chemokines as trophozoites but also other types of chemokines (**Figure 4**). If we look at the same timepoints in the parasites different HCMPs are among the most up-regulated genes (**Figure 6**). The HCMPs are related to the Variant-specific Surface Proteins (VSPs) which are immuno-dominant, cysteine-rich membrane proteins involved in protease protection and antigenic variation (Serradell et al., 2019). The VSPs form a tight surface layer around the *Giardia* trophozoites and they are recognized by TLR4 receptors of the host cells, inducing immune responses (Serradell et al., 2019). In the most recent and complete genome assembly of *Giardia* we identified 133 complete VSP genes and 116 HCMPs (Xu et al., 2020a). This study, combined with our earlier study of trophozoite expression during Caco-2 interaction (Peirasmaki et al., 2020), show that these two gene families are differentially regulated during host cell interactions. The VSPs are the highly expressed genes in *Giardia* and single cell analyses show that one particular VSP transcript dominate per cell (Onsbring et al., 2020). During host cell interactions the VSP expression is not up-regulated, rather a down-regulation of expression is seen (**Table S4**). At the same time, several members of the related HCMP gene family are up-regulated (**Figure 6**). Certain HCMPs are highly up-regulated in both trophozoites and encysting cells (7715, 115066 and 137727) whereas many other HCMPs are mainly up-regulated in the encysting cells (16936, 21321, 91707, 114888, 114991, 115158). The HCMPs studied so far are surface localized (Peirasmaki et al., 2020) and have similar structures in the extracellular domains as VSPs whereas the intracellular domains are different. It is possible that certain HCMPs replace VSPs on the parasite surface during host cell interactions and these new surface proteins can interact with pattern recognition receptors like TLR4 and induce a differential chemokine expression, in turn leading to differential immune responses.

Our data show that the host and parasite responses are dependent on the life-cycle stage of the parasite and earlier it has been shown to be dependent on parasite genotype (Ma’ayeh et al., 2018) and host cell types (Ma’ayeh & Brook-Carter, 2012; Ferella et al., 2014; Holthaus et al., 2021). Thus, the host-parasite responses during interactions are complex and should be studied further in order to fully understand the molecular pathogenesis of *G. intestinalis*. In this study we have used the human colon cancer cell line Caco-2 as host cells. It is the most widely used human enterocytic cell model and it has been used in most studies of *Giardia*-host cell interactions (Emery-Corbin et al., 2020). The Caco-2 cells can easily be differentiated *in vitro* to small intestinal-like enterocytes morphologically and functionally and they are able to express apical brush borders, tight junctions, form mucus layers and express intestinal efflux and uptake transporters. Gene expression in differentiated Caco-2 cells is relatively similar to gene expression in human cells from the duodenum (Lenaerts et al., 2007), which is good in this type of gene expression studies performed here. However, there are differences in certain proteins (Lenaerts et al., 2007) and the gene expression in Caco-2 cells is highly sensitive to changes in culture conditions, clone of Caco-2 and passage number, making it difficult to compare data between different laboratories (Hayeshi et al., 2008). *Giardia* trophozoites are mainly found in the upper small intestine but encysting cells and cysts are found in the lower part of the intestinal system. One interesting observation in this study is that *Giardia* cysts and encysting cell/trophozoite cell debris induce a very strong response in the Caco-2 cells and this can reflect that *Giardia*, even if it is mainly colonizing the duodenum, can give effects on host responses in the lower part of the intestinal region and this has been seen in earlier *in vivo* studies (Cotton et al., 2014; Allain & Buret, 2020). New systems using non-transformed cells are needed in order to further study interactions between *Giardia* and host cells and the recent publication of two studies of *Giardia*-host cell interactions using enteroids derived from human intestinal stem cells show that this is on the way (Holthaus et al., 2021; van Rijn et al., 2022). In these systems it will be important to include parasites or antigens from all life cycle stages of *Giardia* in order to get a complete picture of *Giardia*’s molecular pathogenesis.

## Data Availability Statement

The datasets presented in this study can be found in online repositories. The names of the repository/repositories and accession number(s) can be found below: https://www.ncbi.nlm.nih.gov/geo/, GSE144004, https://www.ncbi.nlm.nih.gov/geo/, GSE144001, https://www.ncbi.nlm.nih.gov/geo/, GSE172213.

## Author Contributions

LR and JG performed the experiments, analyzed the data, and wrote parts of the manuscript. SS conceived and designed the experiments and wrote the first draft of the manuscript. All authors contributed to the article and approved the submitted version.

## Funding

This study was supported by a grant from Vetenskapsrådet-MH (2020-02918) to SS. The funders had no role in study design, data collection, and analysis, decision to publish, or preparation of the manuscript.

## Conflict of Interest

Author SM was employed by Olink Proteomics.

The remaining authors declare that the research was conducted in the absence of any commercial or financial relationships that could be construed as a potential conflict of interest.

## Publisher’s Note

All claims expressed in this article are solely those of the authors and do not necessarily represent those of their affiliated organizations, or those of the publisher, the editors and the reviewers. Any product that may be evaluated in this article, or claim that may be made by its manufacturer, is not guaranteed or endorsed by the publisher.
