# Supplementary material for: Dual RNA Sequencing Reveals Key Events When Different Giardia Life Cycle Stages Interact With Human Intestinal Epithelial Cells In Vitro
Source: Front Cell Infect Microbiol. 2022 Apr 27;12:862211. doi: 10.3389/fcimb.2022.862211 (PMC9094438; doi:10.3389/fcimb.2022.862211)
Supplement: Supplementary file 1 [file DataSheet_1.pdf]

Supplementary figures

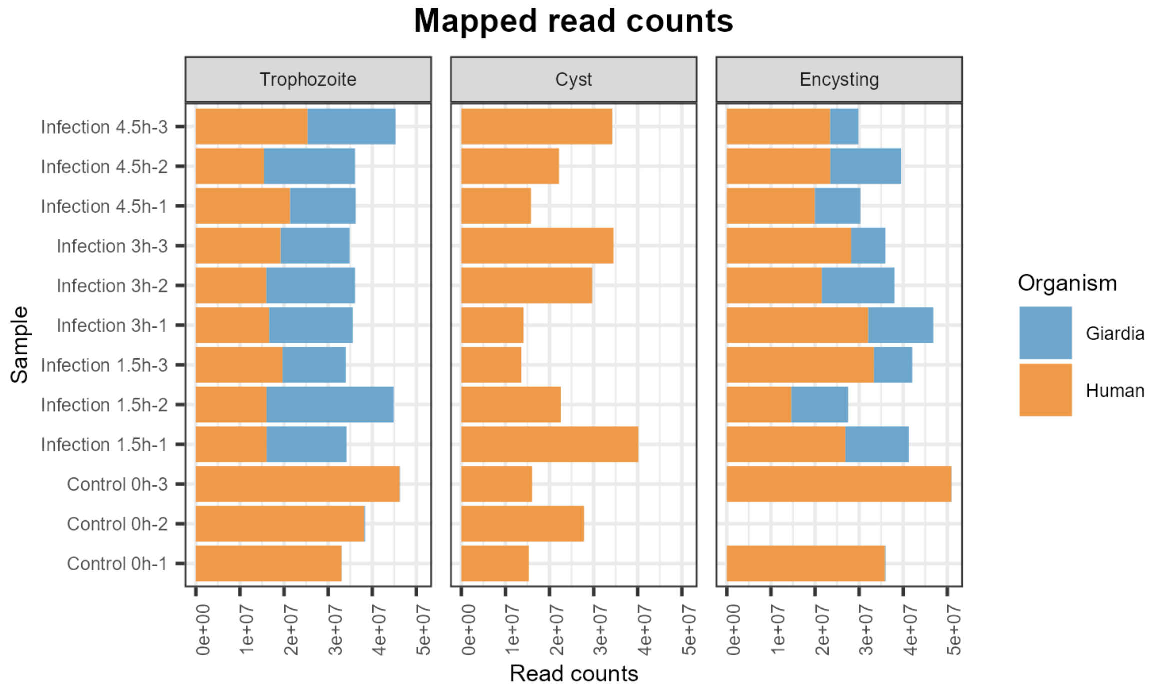

Supplementary Figure 1. Mapped read counts of the three RNA sequencing datasets of differentiated human Caco-2 cell infections with *Giardia* trophozoite, cyst and encysting cells, respectively. Read counts were mapped either to the human or *Giardia* reference genome.

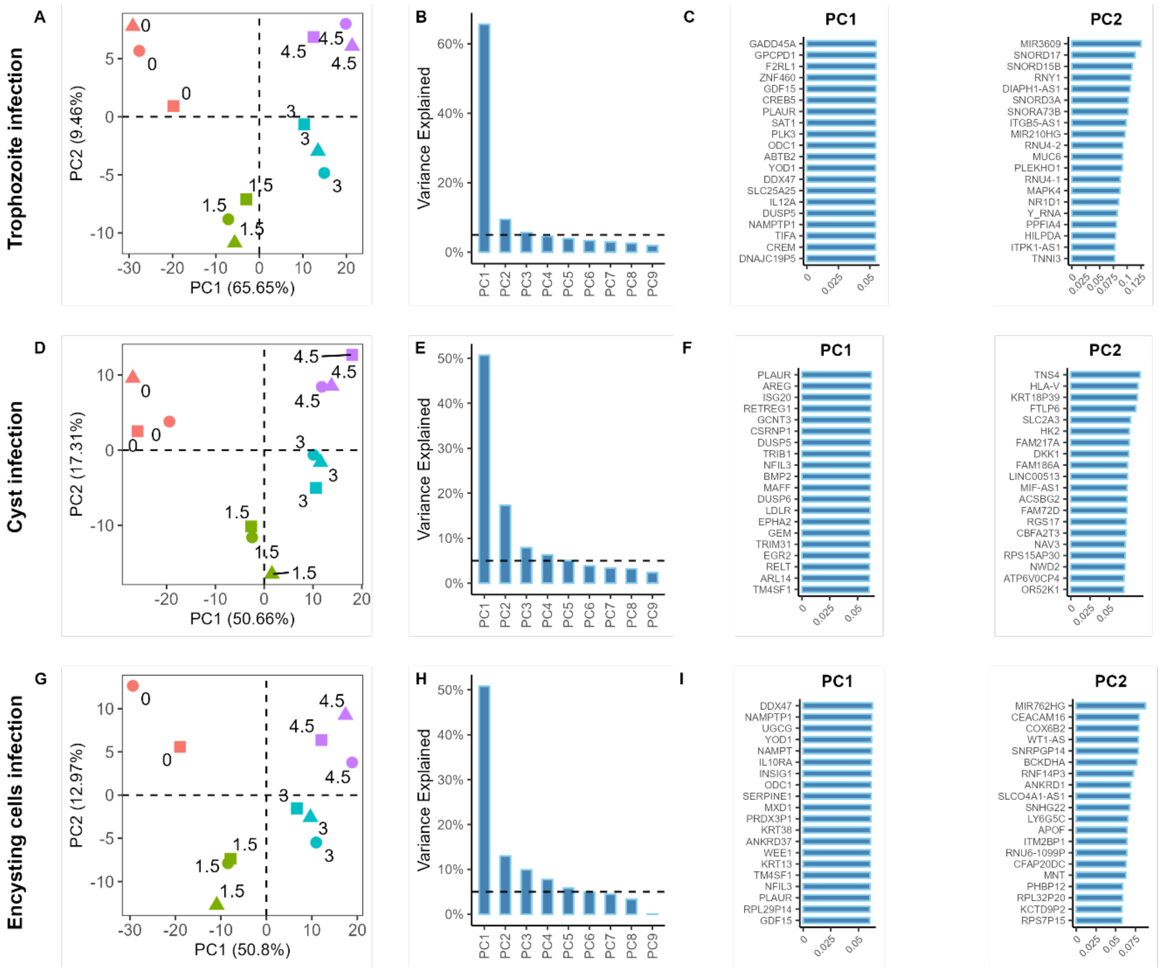

Supplementary Figure 2. Principal Component Analysis (PCA) of the top 500 varying human genes of the RNA sequencing data of differentiated Caco-2 infections with *Giardia* trophozoites, encysting cells and cysts at time point 1.5h, 3h, 4.5h post infection. **A, D, G.** PCA plots of the first two principle components (PCs) of trophozoite, cyst and encysting cells infection, respectively. Variance explained by the respective PC is indicated in brackets on the axis. The sample replicates are separated by shape and the infection time-points by colour. **B, E, H.** Bar plots illustrating the percentage of variance explained by each principle component (PC) in the trophozoite, cyst and encysting cells infections, respectively. The dashed black line indicates 5% explained variance **C, F, I.** Bar plots of the top 30 leading genes in PC1 and PC2 contributing to the variance in the trophozoite, cyst and encysting cells infections, respectively.

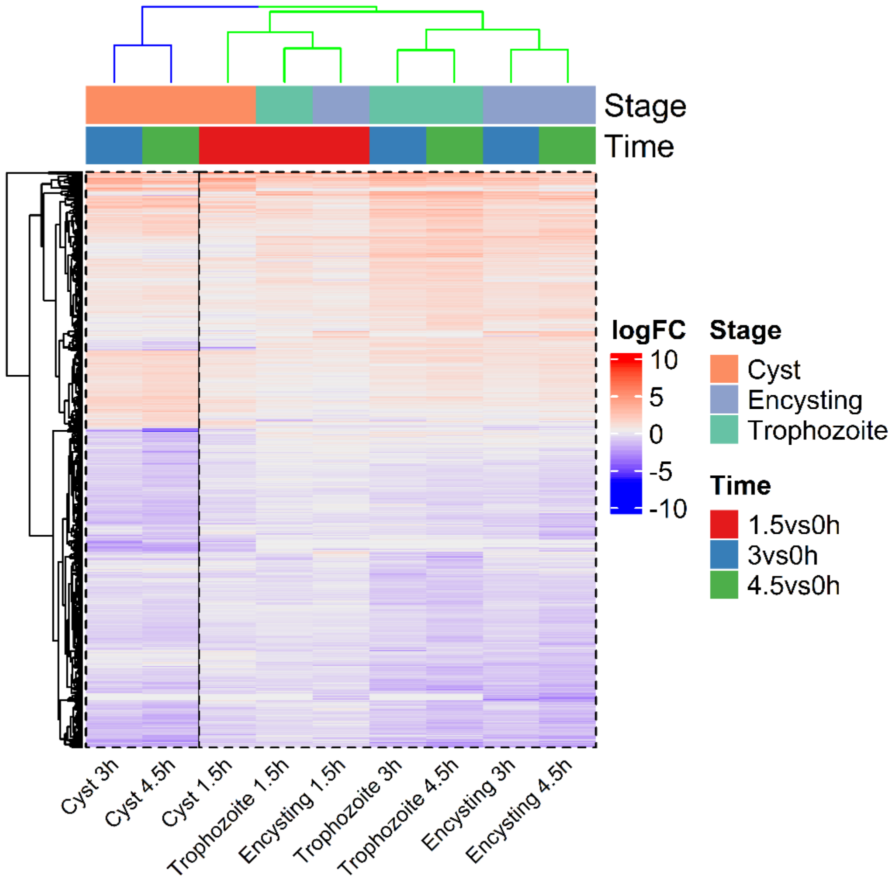

Supplementary Figure 3. Hierarchical clustering of samples of the three RNA sequencing datasets: differentiated human Caco-2 cell infections with trophozoites, encysting cells and cysts. LogFC values of Caco2 genes that were clustered (Complete method, Euclidean distance) if they were significantly differentially transcribed, FDR value<0.05, and if the log_2-fold change value was greater a smaller than one in at least one time point in the three datasets. Insignificant or missing values were set to zero. PVClust cluster analysis and bootstraping (n=10000) showed that there were two significant clusters (approximately unbiased (AU) >= 95%) indicated in the dendogram of the samples with blue or green colours. The AU *p*-values, shown as a percentage, are indicated in red numbers in the dendogram. logFC, log_2-fold change; AU, approximately unbiased

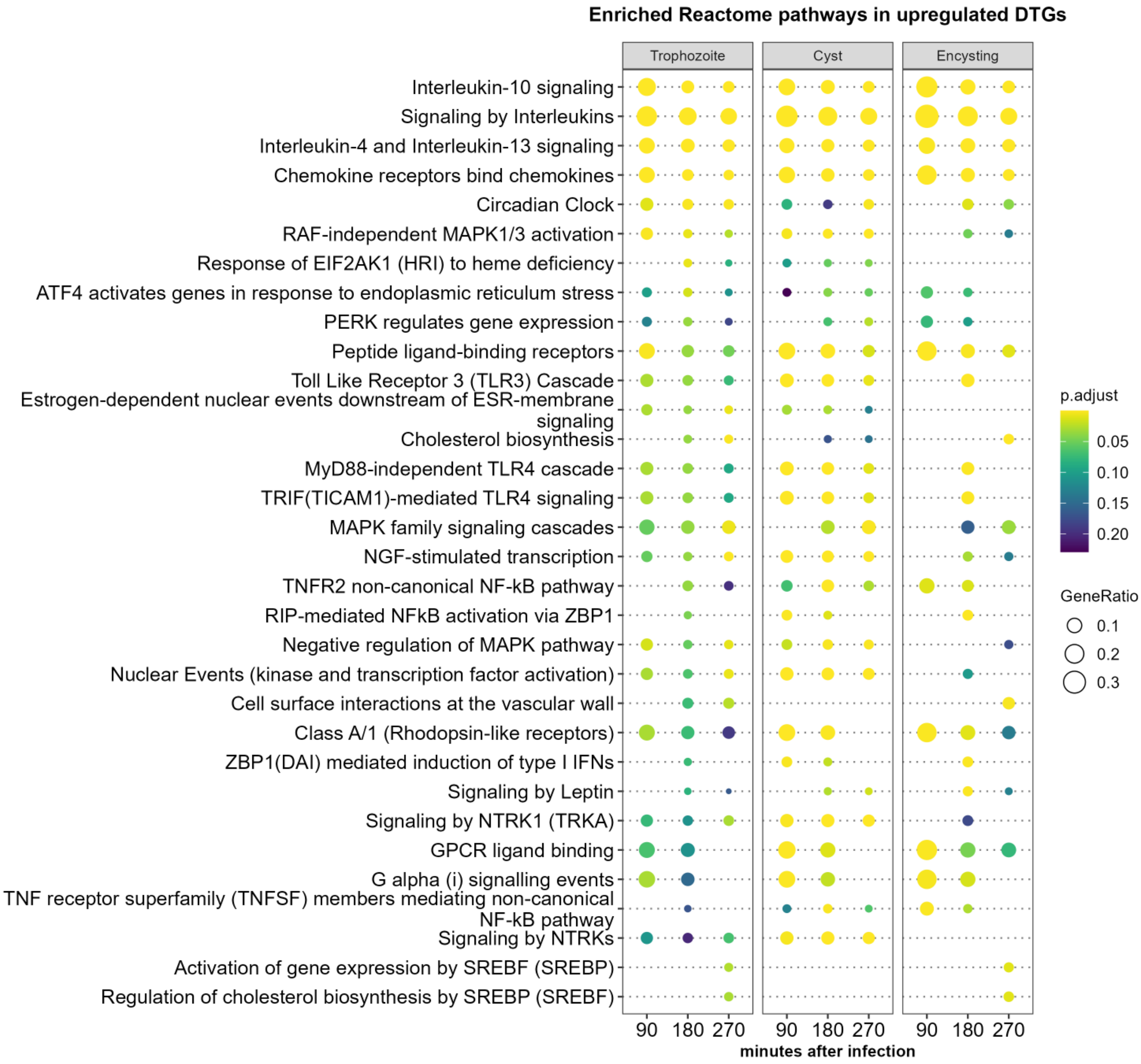

Supplementary Figure 4. Reactome pathway enrichment analysis of up-regulated human genes (more than twofold) during *Giardia* infections of differentiated Caco-2 cells. Comparison of the top 10 enriched Reactome pathway terms in each of the different *Giardia* stage infections (trophozoite, cyst and encysting cells Caco-2 infections) and infection timepoints (1.5, 3, and 4.5 h post-infection). Circle size and colour indicate the gene ratio (number of differentially expressed genes against the number of genes associated with an enrichment term) and their significance, respectively.

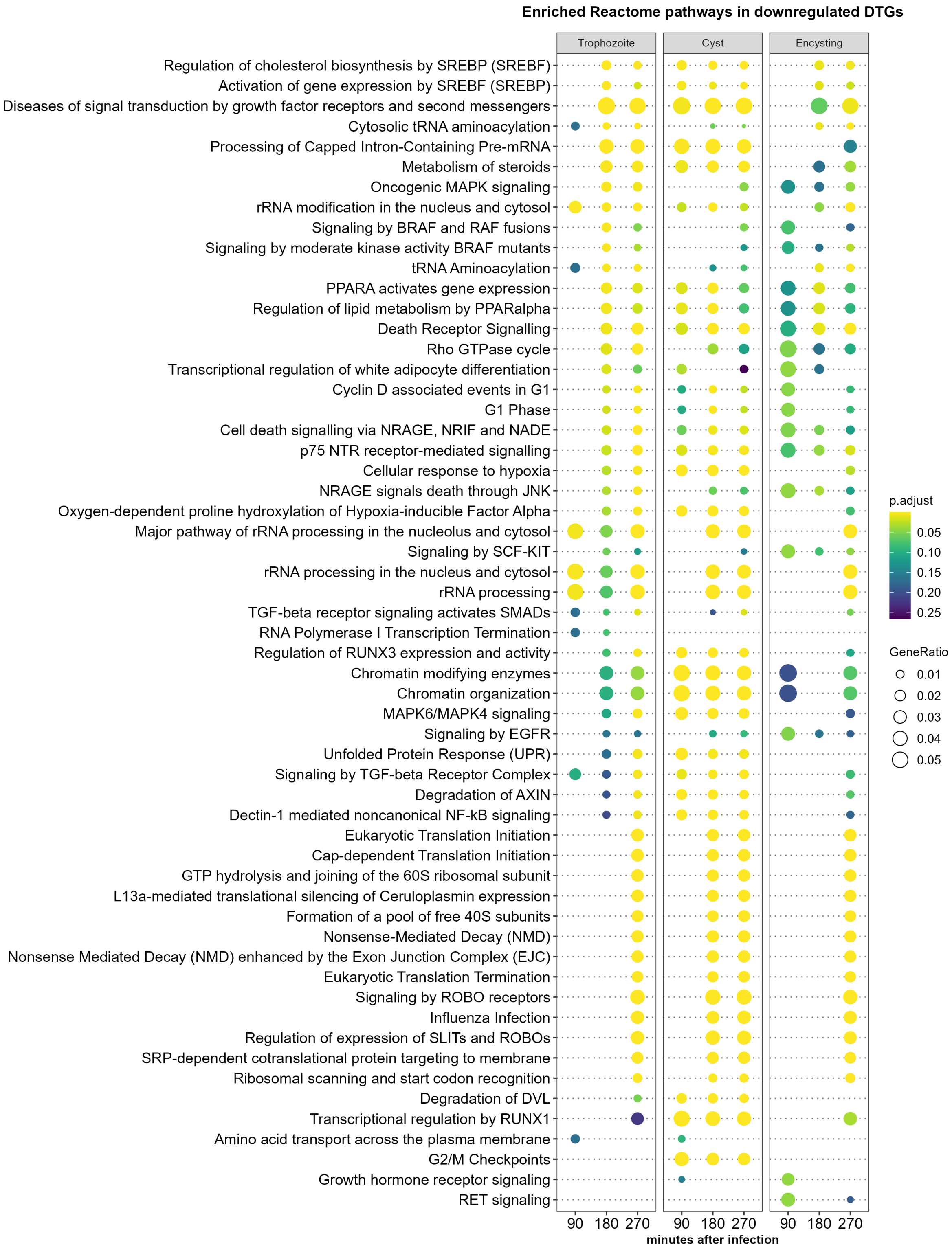

Supplementary Figure 5. Reactome pathway enrichment analysis of down-regulated human genes (more than two-fold) during *Giardia* infections of differentiated Caco-2 cells. Comparison of the top 10 enriched Reactome pathway terms in each of the different *Giardia* stage infections (trophozoite, cyst and encysting cells Caco-2 infections) and infection timepoints (1.5, 3, and 4.5 h post-infection). Circle size and colour indicate the gene ratio (number of differentially expressed genes against the number of genes associated with an enrichment term) and their significance, respectively.

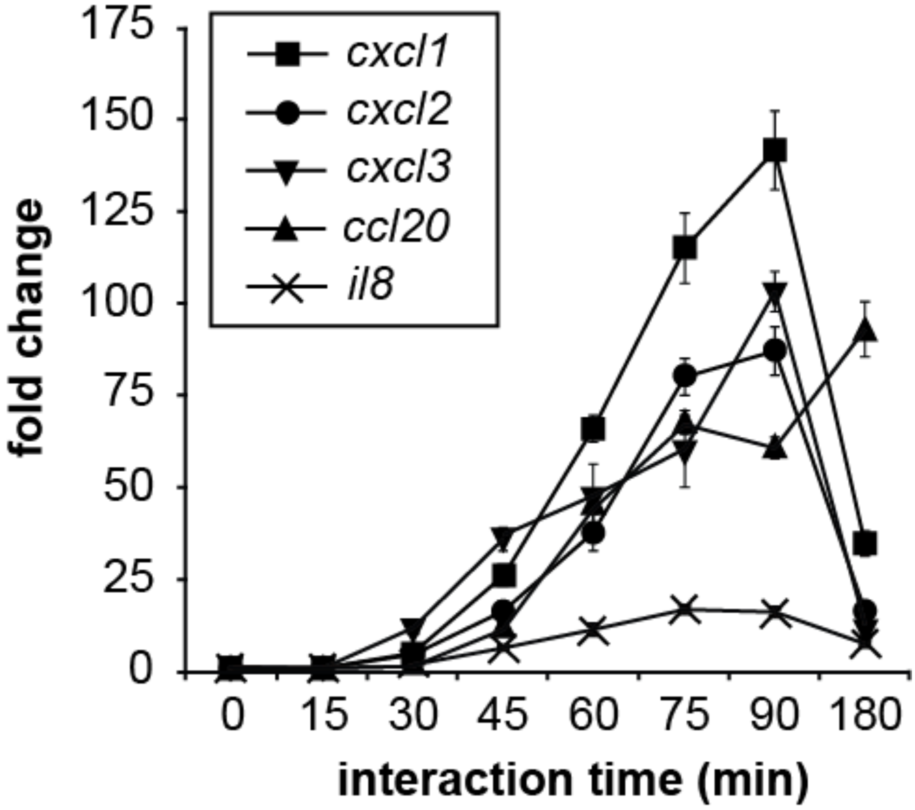

Supplementary Figure 6. RT-PCR analyses of chemokine expression in differentiated Caco-2 cells during interaction with *G. intestinalis* WB trophozoites.

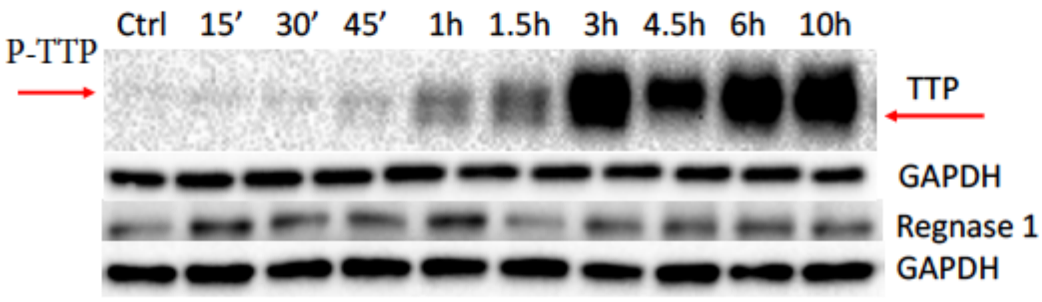

Supplementary Figure 7. Western blot analyses of human proteins expressed in differentiated Caco-2 cells during interactions with trophozoites. TTP and Regnase-1 expression in the Caco-2 cells between 15 min and 10 h of interaction. Arrows indicate phosphorylated TTP (P-TTP) and non-phosphorylated TTP (TTP). GAPDH constitutive loading control.

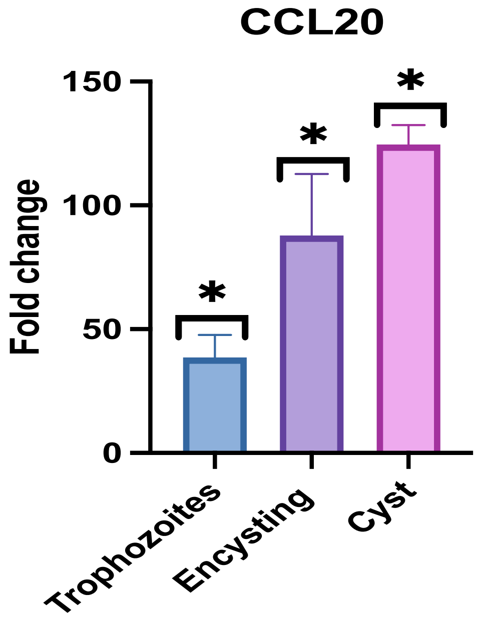

Supplementary Figure 8. RT-PCR analyses of chemokine expression of CCL20 in differentiated Caco-2 cells during interaction with *G. intestinalis* WB trophozoites, 7 h encysting cells and cysts.

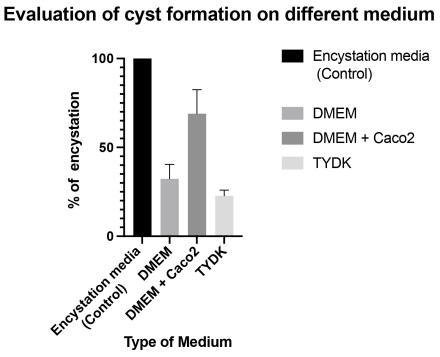

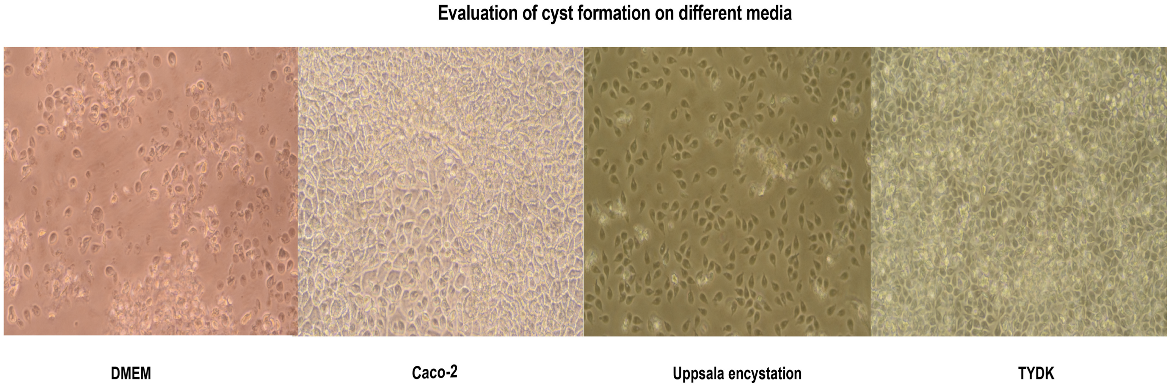

Supplementary Figure 9. Encystation of *G. intestinalis* WB-C6 7 h encysting parasites in different conditions. The trophozoites were encysted in standard Uppsala encystation medium for 7 h and then added to 4 different conditions for another 30 h: DMEM, Differentiated Caco-2 cells with DMEM, Uppsala encystation medium and TYDK- trophozoite growth medium. Pictures from the cultures in the end of the experiment showing cysts and attached trophozoites.
